# Directed Biosynthesis of New to Nature Alkaloids in a Heterologous *Nicotiana benthamiana* Expression Host

**DOI:** 10.3389/fpls.2022.919443

**Published:** 2022-06-22

**Authors:** Marianna Boccia, Dagny Grzech, Adriana A. Lopes, Sarah E. O’Connor, Lorenzo Caputi

**Affiliations:** ^1^Department of Natural Product Biosynthesis, Max Planck Institute for Chemical Ecology, Jena, Germany; ^2^Biotechnology Unit, Universidade de Ribeirão Preto (UNAERP), Ribeirão Preto, Brazil

**Keywords:** natural product, directed biosynthesis, new-to-nature products, *Nicotiana benthamiana*, monoterpene indole alkaloid, alstonine, stemmadenine

## Abstract

Plants produce a wide variety of pharmacologically active molecules classified as natural products. Derivatization of these natural products can modulate or improve the bioactivity of the parent compound. Unfortunately, chemical derivatization of natural products is often difficult or impractical. Here we use the newly discovered biosynthetic genes for two monoterpene indole alkaloids, alstonine and stemmadenine acetate, to generate analogs of these compounds. We reconstitute these biosynthetic genes in the heterologous host *Nicotiana benthamiana* along with an unnatural starting substrate to produce the corresponding new-to-nature alkaloid product.

## Introduction

*Nicotiana benthamiana*, a plant belonging to the *Solanaceae* family, has been widely used as a system for synthetic biology and metabolic engineering efforts. A notable application of *N. benthamiana* includes the reconstitution of metabolic pathways that yield high value small molecules (specialized metabolites or natural products) through transient transformation (Akhtar et al., 2011; Arntzen, 2015; Bally et al., 2015; Liu, 2022). *N. benthamiana* has, to date, been employed for the production of many different plant specialized metabolites including alkaloids (Miettinen et al., 2014), betalains (Polturak et al., 2016), terpenoids (Reed and Osbourn, 2018), glucosinolates (Crocoll et al., 2016), and cyanogens (Rajniak et al., 2015). In this approach, leaves of young *N. benthamiana* plants are infiltrated with a strain of *Agrobacterium tumefaciens* that harbors the gene of interest in a plant expression construct. To express multi-gene pathways, a leaf of an intact *N. benthamiana* plant is simultaneously co-infiltrated with different *A. tumefaciens* strains, where each *A. tumefaciens* strain harbors a different gene in the desired expression construct. Heterologous protein produced in the infected *N. benthamiana* leaf reaches a peak between 5- and 7- days after the infiltration, resulting in the production of the pathway metabolite (LeBlanc et al., 2021). *N. benthamiana* is an ideal host in which to reconstitute plant derived metabolic pathways, since this plant host contains all cofactors, intracellular compartments, and protein folding machinery required for successful production of plant proteins (Reed and Osbourn, 2018).

**FIGURE 1:**
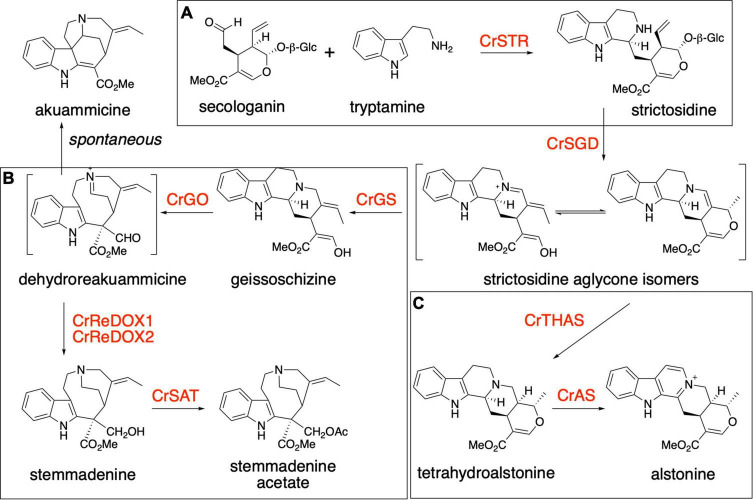
Biosynthesis of monoterpene indole alkaloids. **(A)** Production of strictosidine from tryptamine and secologanin by the action of *Catharanthus roseus* strictosidine synthase (CrSTR). **(B)** Biosynthesis of stemmadenine acetate, an intermediate of the pathway for the production of the two anticancer compounds vinblastine and vincristine. The production of stemmadenine acetate involves 6 enzymes from strictosidine: CrSGD, CrGS, CrGO, CrReDOX1, CrReDOX2, and CrSAT. Akuammicine is a spontaneous degradation product of preakuammicine. **(C)** Biosynthesis of the antipsychotic molecule alstonine in *Catharanthus roseus.* The production of alstonine involves 3 enzymes from strictosidine: CrSGD, CrTHAS, CrAS.

The plant *Catharanthus roseus* (*Apocynaceae*) is a medicinal plant that produces over 100 specialized metabolites classified as monoterpene indole alkaloids (MIAs). MIAs have diverse biological activities, including anti-cancer (vinblastine and vincristine), anti-psychotic (alstonine), anti-hypertensive (ajmaline) and anti-malarial (quinine) (van Der Heijden et al., 2004; Elisabetsky and Costa-Campos, 2006; Achan et al., 2011; Monasky et al., 2021). The universal MIA precursor in plants is strictosidine, a biosynthetic intermediate that is formed via an enzyme-catalyzed (strictosidine synthase, CrSTR) Pictet-Spengler condensation of the monoterpenoid secologanin and amino acid derived tryptamine (Figure 1A; Mizukami et al., 1979; Treimer and Zenk, 1979). The deglycosylation of strictosidine, catalyzed by strictosidine glucosidase (CrSGD), is the starting point for the biosynthesis of anti-cancer drug vinblastine. The biosynthetic pathway of vinblastine, which comprises ca. 30 enzymatic steps, proceeds from strictosidine aglycone through a series of unstable intermediates to form the central biosynthetic precursor, stemmadenine acetate (Supplementary Figure 1). Strictosidine aglycone is acted upon by a medium-chain alcohol dehydrogenase (MDR), geissoschizine synthase (CrGS) to generate geissoschizine. This is oxidized to dehydropreakuamicine by a cytochrome P450, geissoschizine oxidase (CrGO). The MDR CrRedox 1 and the aldo-keto reductase CrRedox2 (Tatsis et al., 2017; Qu et al., 2018) reduce dehydropreakuamicine to stemmadenine and then finally an acetyl transferase (stemmadenine acetyltransferase, Cr SAT) generates stemmadenine acetate (Qu et al., 2018; Figure 1B). A variety of alkaloids, including the direct precursors of vinblastine, catharanthine and tabersonine, are generated from stemmadenine acetate. In the case of catharanthine and tabersonine biosynthesis, stemmadenine acetate is oxidized (PAS), reduced (DPAS) and then cyclized (CS or TS) (Supplementary Figure 1). Moreover, in addition to the vinblastine biosynthetic pathway, *C. roseus* produces over 100 additional MIA that derive from strictosidine aglycone. The compound alstonine is generated by reduction of strictosidine aglycone (Shamma and Richey, 1963) to tetrahydroalstonine through the action of the MDR tetrahydroalstonine synthase (CrTHAS) (Stavrinides et al., 2015, 2016) and then converted to alstonine by a cytochrome P450, alstonine synthase (CrAS) (Dang et al., 2018; Figure 1C). Alstonine has antipsychotic (Costa-Campos et al., 1999; Elisabetsky and Costa-Campos, 2006) and antiplasmodial (Arnold et al., 2021) activities.

After establishing a system for biosynthetic pathway reconstitution, a variety of metabolic engineering strategies can be used to modify the biosynthetic pathway to produce “un-natural” or “new-to-nature” compounds. We have previously employed *N. benthamiana* as a heterologous host for the production of simple tryptophan-derived new-to-nature auxin analogs by generating plant-derived auxin biosynthetic enzymes in combination with bacterial halogenases (Davis et al., 2020). Here, we reconstitute the biosynthetic pathways of both stemmadenine acetate and alstonine in the plant *N. benthamiana* to establish a platform for the production of analogs of these compounds via precursor-directed biosynthesis starting from un-natural substrate analogs. We generated analogs of strictosidine *in vitro* using tryptamine derivatives (fluoro-, chloro-, bromo-, methyl-, methoxy, hydroxyl-tryptamines) as starting material and the Pictet-Spenglerase CrSTR as catalyst. These analogs were then purified and co-infiltrated into *N. benthamiana* along with the biosynthetic genes for either stemmadenine acetate (CrSGD, CrGS, CrGO, CrReDOX1, CrReDOX2, CrSAT) or alstonine (CrSGD, CrTHAS, CrAS). This work demonstrates that it is possible to use directed biosynthesis in combination with heterologous reconstitution in *N. benthamiana* to produce new-to-nature complex alkaloid analogs.

## Materials and Methods

### CrSTR Expression and Purification

A truncated version of CrSTR, in which the native vacuolar signal peptide (first N-terminal 21 amino acids) was removed, was cloned into pOPINA vector (Addgene #41141) and transformed into *E. coli* strain SoluBL21 for protein expression. A single colony grown in LB agar media supplemented with kanamycin (50 μg/mL) was grown overnight in 10 mL 2xYT media at 37°C. The next day, 1 mL of the overnight culture was used to inoculate 1 L of 2xYT media. The fresh culture was grown at 37°C until OD_600_ of 0.6–0.8 was reached, followed by induction with 300 μM IPTG and incubation with 220 rpm shaking speed at 18°C for 16–18 h. The next day, the cells were centrifuged for 15 min at 4,000 *g*, resuspended in 20 mL of binding buffer (50 mM tris-HCl pH 8, 50 mM glycine, 500 mM sodium chloride, 20 mM imidazole, 5% v/v glycerol, pH 8) containing in addition 0.5 mg/mL Lysozyme and EDTA free protease inhibitor cocktail (Roche cOmplete™). Cells were lysed by pulsed sonication for 4 min. The crude lysates were centrifuged at 38,000 *g* for 30 min and the lysate filtered through a 0.45 μm filter.

His_6_-tagged enzyme purification was performed on a AKTA pure system (GE Healthcare) using a HisTrap HP 5 mL column (GE Healthcare) equilibrated with Buffer A. Samples were loaded at a flow rate of 2 mL/min and step-eluted using Buffer B (50 mM Tris-HCl pH 8, 50 mM glycine, 500 mM NaCl, 5% glycerol, 500 mM imidazole). The protein was dialyzed in Buffer A4 (20 mM HEPES pH 7.5; 150 mM NaCl) in centrifugal concentrators with size exclusion of 10 KDa. Proteins were aliquoted in 50 μL Eppendorf tubes, flash-frozen and stored at –80°C.

### One-Pot Enzymatic Reaction for the Synthesis of Strictosidine and Its Analogs and Purification by Preparative HPLC

Secologanin (4 mM) and tryptamine analogs (6 mM) obtained from different suppliers (Sigma Aldrich, Cayman Chemical, Biosynth, Fluorochem, Alfa Aesar, TRC), as in Supplementary Table 1, were combined in a total volume of 50 mL of phosphate buffer (100 mM potassium phosphate, pH 7.8). CrSTR was added to the 10 mL reaction with a final concentration of 2.5 μM, and the reaction was stirred at room temperature for 24 h. Strictosidine analogs were pre-purified using a 500 mg reverse-phase SPE column (Discovery DSC-18, Supelco). The column was activated with 3 mL of MeOH, equilibrated with 3 mL of water before loading the sample. After wash with water (3 mL), the strictosidine analogs were eluted with 1 mL of MeOH. Samples were then subjected to preparative HPLC (Agilent 1260 Infinity II) using a Luna-C18 column (5 μm, 100 A, 250 × 30 mm) connected to an Agilent 1290 Infinity II for collection. The solvents used were solvent A (water containing 0.1% formic acid) and B (acetonitrile with 0.1% formic acid). The flow rate was set at 30.0 mL/min and the gradient was 10% B from 0 to 28 min, 50% B from 28.1 to 30 min, 10% B from 30.1 to 35 min. The fractions were collected and evaporated to dryness using a Genevac EZplus.

### Transient Expression in *Nicotiana benthamiana*

The genes CrTHAS and CrAS (Supplementary Table 2) were amplified from *C. roseus* (Sunstom Apricot) cDNA prepared using the SuperScript VILO (Thermo Fisher Scientific). Synthetic genes CrSGD, CrGS, CrGO, CrRedOx1, CrRedOx2 and CrSAT domesticated for the *Bsa*I site (Supplementary Table 2) were purchased from Twist Bioscience^1^. All the genes were amplified using the primers with specific over-hangs (Supplementary Table 3) by employing Phusion High-Fidelity DNA Polymerase (New England Biolabs) according to the manufacturer’s instructions. The *Sl*Ubi10 promoter and terminator were amplified from pUPD plasmids. The PCR products were purified from agarose gel and ligated into 3α1 vector via GoldenBraid 3.0 cloning (*Bsa*I and T4 ligase were from NEB) (Vazquez-Vilar et al., 2017).

The constructs were used to transform *E. coli* Stellar cells by heat shock, plated on selective LB medium containing kanamycin (50 μg/mL). The resulting colonies were screened by colony PCR using the appropriate primers (Supplementary Table 3) and Sanger sequenced to confirm the correct insertion of the genes. Aliquots of competent *A. tumefaciens* cells (GV3101) were transformed by electroporation using 2 μL of each plasmid (100–200 ng of DNA) for each gene of interest. After electroporation, cells were recovered for 3 h at 28°C in selective LB medium and plated on LB-agar media with selective antibiotics (rifampicin 50 μg/mL, gentamicin 25 μg/mL, kanamycin 50 μg/mL) for 48 h at 28°C.

### Transient Expression in *Nicotiana benthamiana* and Metabolite Extraction

Single colonies of *A. tumefaciens* were grown in 10 mL of LB supplemented with antibiotics as described above for 48 h at 28°C with shaking at 200 rpm. The cultures were then centrifuged at 4,000 rpm for 20 min and re-suspended in 10 mL of infiltration buffer (10 mM NaCl, 1.75 mM CaCl_2_, 100 μM acetosyringone) and left at room temperature for 2 h. The OD was measured and the cultures were diluted until the OD at 600 nm of each was adjusted to 0.3. Six individual *A. tumefaciens* cultures, with each one harboring an individual stemmadenine acetate metabolic pathway gene (CrSGD, CrGS, CrGO, CrReDOX1, CrReDOX2, and CrSAT) were mixed and 2 mL of solution with a final OD of 0.3 was used to infiltrate two leaves of 3–4 week-old *N. benthamiana* plants using a 1 mL syringe without needle. Analogously, three individual *A. tumefaciens* cultures, with each one harboring an alstonine metabolic pathway gene (CrSGD, CrTHAS and CrAS) were mixed and used to infiltrate two leaves for each *N. benthamiana* plant. After 5 days, the desired strictosidine analog substrate dissolved in infiltration buffer (2 mL per leaf, 200 μM) was infiltrated into the transfected leaves in the same way. The leaves were harvested after 48 h, flash-frozen in liquid nitrogen and stored at –80°C until extraction.

The extraction of metabolites was performed on 150 mg of each powdered frozen leaf sample by adding 300 μL of 80% MeOH + 0.1% formic acid and sonicating the samples in a water bath (25°C) for 10 min. Samples were incubated for 1 h at room temperature and centrifuged for 10 min at top speed in a table-top centrifuge. The supernatant was filtered through PTFE (0.2 μm) and analyzed by UHPLC-MS/MS. Unless stated otherwise, at least three biological replicates were used for the metabolite analysis.

### Metabolite Analysis by Ultra-Performance Liquid Chromatography-Tandem Mass Spectrometry

Metabolites were analyzed by ultra-performance liquid chromatography-Tandem mass spectrometry (UHPLC-MS/MS) on a UHPLC system (Elute LV, Bruker) connected to a triple quadrupole (EVOQ, Bruker) mass spectrometer. Chromatography was performed using a Phenomenex Kinetex XB-C18 column (2.1 × 100 mm, 2.6 μm) column kept at 40°C. Water containing 0.1% formic acid and acetonitrile containing 0.1% formic acid were used as mobile phases A and B, respectively, with a flow rate of 0.6 mL/min. The gradient was 10% B from 0 to 6 min, 30% B from 6 to 6.1 min, 100% B from 6.10 to 7.50 min and 10% B from 7.6 to 11 min. The analysis was carried out in positive mode (ESI) and the samples were kept at 10°C. The injection volume of both the standard solutions and the samples was 2 μL. Capillary voltage was 3,500 V; the source was kept at 450°C; cone temperature was 350°C; cone gas flow 20 L/h; and nebulizer gas flow, 50 L/h. Unit resolution was applied to each quadrupole. Flow injections of serpentine, stemmadenine acetate, tetrahydroalstonine and akuammicine were used to optimize the multiple reaction monitoring (MRM) conditions. The cone voltage was experimentally determined and the collision energies were automatically adjusted by the MS Workstation software 8.2 (Bruker). A dwell time of at least 25 ms was applied to each MRM transition. For the detection and quantification of analogs for which authentic standards were not available, MRM signals were predicted based on the fragmentation patterns and collision energies (Supplementary Table 4) of the parent standards.

High resolution and MS/MS analysis of the halogenated analogs produced in *N. benthamiana* was performed on a qExactive Plus mass spectrometer (Thermo) coupled to a Vanquish UPLC system (Thermo). Chromatography was performed on a Kinetex XB-C18 column with 2.6 μμm particle size and dimensions of 50 mm x 1 mm (Phenomenex) kept at 40°°C. Mobile phases were: A = 0.1% formic acid and B = acetonitrile. The gradient was 10% B at time 0 and until 1 min, linear gradient from 1 min to 6 min up to 30% B, at 6.1 min 100% B until 7.9 min, then column re-equilibration at 10% B until time 10 min. The flow rate was 0.3 mL/min and the injection volume was 2 μL.

The mass spectrometer was operated in positive electrospray mode. The source parameters were: Spray voltage 3.5 kV, Capillary temperature 250°C, gas temperature 400°C. Data were acquired in Parallel reaction Monitoring (PRM) mode in which the instrument acquires MS/MS scans based on a specified inclusion list. The quadrupole isolates the precursor ions, which are then sent to the C-Trap and transmitted to the HCD cell for fragmentation. The fragmented ions return to the C-Trap and are subsequently injected into the Orbitrap mass analyzer. The inclusion list was constituted of the following masses: *m/z* 349.15467 (C_21_H_20_N_2_O_3_) for alstonine and serpentine, *m/z* 367.14525 (C_21_H_19_N_2_O_3_F) F-alstonine, *m/z* 383.11570 (C_21_H_19_N_2_O_3_Cl) for Cl-alstonine, *m/z* 397.21218 for stemmadenine acetate (C_23_H_28_N_2_O_4_) and *m/z* 415.20276 (C_23_H_27_N_2_O_4_F) for F-stemmadenine acetate.

## Results

### Enzymatic Production of Strictosidine Analogs *in vitro*

**FIGURE 2:**
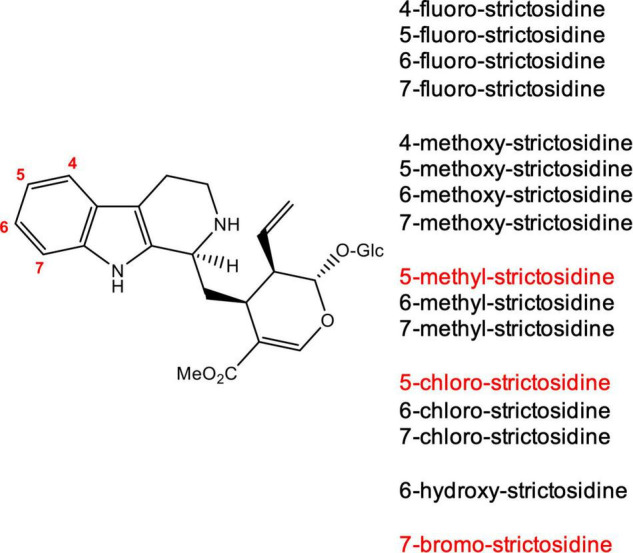
Strictosidine analogs produced *in vitro* using tryptamine analogs and secologanin. The reaction was catalyzed by *Cr*STR. Strictosidine analogs shown in red were not turned over by *Cr*STR or were not produced in high enough levels for isolation.

We generated strictosidine analogs *in vitro* by incubating the corresponding commercially available tryptamine analogs (4-, 5-, 6-, and 7-fluoro tryptamine, 4-, 5-, 6-, and 7-methoxy tryptamine, 6- and 7-chloro tryptamine, 6- and 7-methyl tryptamine and 6-hydroxy tryptamine) with secologanin and the enzyme CrSTR (Figure 2). When incubated under the same conditions, the analogs 5-chloro, 5-methyl and 7-bromo tryptamine were not converted or converted in very low quantities that could not be isolated. The substrate specificity of strictosidine synthase has been well-established, and our results were consistent with these previous reports (McCoy et al., 2006; Loris et al., 2007; Eger et al., 2020; Sheng and Himo, 2020). After purification by preparative HPLC, the 13 strictosidine analogs obtained were used as a starting substrate for the production of the corresponding alstonine and stemmadenine acetate analogs.

### Production of Fluorinated and Chlorinated Analogs of Alstonine in *Nicotiana benthamiana*

Co-infiltration of the bacteria harboring the plasmids for the expression of the genes involved in the production of alstonine (CrSGD, CrTHAS and CrAS), along with one of the strictosidine analogs generated as described above, in 4-week-old *N. benthamiana* plants led to the formation of 4-fluoro-alstonine (Supplementary Figure 2), 5-fluoro-alstonine (Supplementary Figure 3), 6-fluoro-alstonine (Supplementary Figure 4), 7-fluoro-alstonine (Supplementary Figure 5) and 7-chloro-alstonine (Supplementary Figure 6) *in vivo*. The identity of the products was assigned based on exact mass of the compound and comparison of their fragmentation pattern with serpentine, a commercially available diastereomer of alstonine, that shows the same fragmentation spectrum of alstonine (Supplementary Figure 8).

No conversion to the corresponding alstonine analog was observed for any of the methoxy, methyl- and hydroxy-strictosidine analogs that we tested (Table 1). For methyl and methoxy strictosidine analogs, we observed formation of a compound with a mass corresponding to the immediate precursor to alstonine, tetrahydroalstonine (Supplementary Figure 8). This suggests that CrSGD (Yerkes et al., 2008) and CrTHAS have broad substrate specificity, while the oxidase AS has a more limited substrate scope because of steric and/or electronic incompatibility. For hydroxy-strictosidine, no reduced product was observed.

**TABLE 1:** Production of alstonine and stemmadenine analogs from strictosidine analogs in *N. benthamiana* following the co-infiltration of the three genes involved in the alstonine pathway (CrSGD, CrTHAS, CrAS) or the six genes involved in the stemmadenine pathway (CrSGD, CrGS, CrGO, CrReDOX1, CrReDOX2, CrSAT).

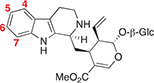	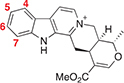	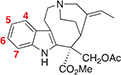
Strictosidine analog	Alstonine analog ng g/FM	Stemmadenine acetate analog
Strictosidine	24.7 ± 2.0	Not quant.
4-Fluoro-strictosidine	27.5 ± 7.1	Not quant.
5-Fluoro-strictosidine	154.7 ± 31.4	Not quant.
6-Fluoro-strictosidine	190.8 ± 21.7	Not quant.
7-Fluoro-strictosidine	122.3 ± 2.1	Not quant.
4-Methoxy-strictosidine	Nd	Nd
5-Methoxy-strictosidine	Nd	Nd
6-Methoxy-strictosidine	Nd	Nd
7-Methoxy-strictosidine	Nd	Nd
6-Methyl-strictosidine	Nd	Nd
7-Methyl-strictosidine	Nd	Nd
6-Chloro-strictosidine	Nd	Nd
7-Chloro-strictosidine	27.2 ± 5.0	Nd
6-Hydroxy-strictosidine	Nd	Nd

*Yields were quantified as ng per gram of fresh plant material (ng g/FM). nd = not detected. not quant. = detected but not quantified. n = 4.*

We estimated the following yields: 28 ng of 4-fluoro-alstonine per g of plant fresh mass (FM), 155 ng of 5-fluoro-alstonine per g of FM, 190 ng of 6-fluoro-alstonine per g of FM, 122 ng of 7-fluoro-alstonine per g of FM and 27 ng of 7-chloro-alstonine per g of FM. Only 25 ng/g of FM of alstonine were accumulated when infiltrating with natural strictosidine (Table 1).

### Production of Fluorinated Stemmadenine Acetate Analogs in *Nicotiana benthamiana*

The production of stemmadenine acetate analogs was achieved by co-infiltrating the bacterial suspensions harboring the plasmids for the expression of the six genes CrSGD, CrGS, CrGO, CrReDOX1, CrReDOX2, and CrSAT in *N. benthamiana* along with strictosidine analogs as starting substrate. The assignment of the identity of the products was made as described above, using the exact mass of the compound and comparison of the fragmentation pattern with an authentic standard of stemmadenine (Supplementary Figure 9). We observed formation of compounds corresponding to fluoro-stemmadenine acetate analogs: 4-fluoro-stemmadenine acetate (Supplementary Figure 10), 5-fluoro-stemmadenine acetate (Supplementary Figure 11), 6-fluoro-stemmadenine acetate (Supplementary Figure 12) and 7-fluoro-stemmadenine acetate (Supplementary Figure 13), confirmed by UHPLC/MS analysis. Levels of production were substantially lower than what was observed for the alstonine pathway and were too low for accurate estimated quantification. These low levels were likely due to the accumulation of the corresponding akuammicine analog, which results from non-enzymatic deformylation of the product of CrGO (Tatsis et al., 2017; Figure 1 and Supplementary Figure 14). Additionally, a fraction of the stemmadenine acetate was oxidized by an endogenous oxidase present in *N. benthamiana*, which further complicated quantification analysis (Caputi et al., 2018; Supplementary Figure 15). No conversion to stemmadenine was observed for the chloro-, methoxy-, methyl- and hydroxy- strictosidine precursors (Table 1). For 6- and 7-methyl strictosidine, the corresponding akuammicine derivatives appeared to be the major product, suggesting that CrSGD, CrGS and CrGO can accept these substrates, but downstream enzymes cannot (Supplementary Figure 16). No downstream products were observed for methoxy, hydroxy and chloro-strictosidine (Supplementary Figure 17).

## Discussion

Many analogs of natural products are used in pharmacology and medicine (Guo, 2017). These analogs are often made synthetically or semi-synthetically, but directed biosynthesis provides an alternative method for accessing these compounds (Sun et al., 2015). By adding different functional groups to a molecule, it is possible to modify and improve interactions with the active site of the target protein and alter the physiochemical, pharmacokinetic and pharmacodynamics properties (Jeschke, 2004). Halogenation and chlorination are just two of the modifications that can change the properties of a compound. Indeed, methylation, metoxylation and hydroxylation can alter the activity and pharmacokinetic properties of drugs. Methylation is a modification that can improve potency and the duration of the effect of medicinal drugs (Bazzini and Wermuth, 2008), by modifying solubility, bioavailability, and pharmacokinetics of the compounds (Barreiro et al., 2011). The introduction of a hydroxyl group can change the physicochemical proprieties of a molecule by increasing its polarity, improving the selectivity and the affinity with receptors (Cramer et al., 2019).

Previously, we performed directed biosynthesis to generate analogs of monoterpene indole alkaloids in the native plant producer *C. roseus*. We incubated both seedlings and hairy root cultures of *C. roseus* with tryptamine and strictosidine analogs and observed the incorporation of these precursors into a variety of monoterpene indole alkaloids (McCoy and O’Connor, 2006). Now many of the MIA genes of *C. roseus* have been identified, which allows heterologous expression of these pathways, rather than utilizing the endogenous pathways of the native producer plant. Therefore, we explored whether heterologous reconstitution of two representative *C. roseus* derived monoterpene indole alkaloid pathways in *N. benthamiana* could be used in combination with infiltration of non-natural substrates to generate alkaloid analogs.

The two compounds that we selected for this study were alstonine and stemmadenine acetate. Alstonine has anti-psychotic activity, while stemmadenine acetate is an advanced precursor for two anticancer drugs vinblastine and vincristine. The pathway genes for both of these compounds have been identified. For this study, we first generated analogs of strictosidine *in vitro*, by an enzymatic condensation of secologanin and tryptamine analogs (fluoro-, chloro-, bromo-, methyl-, methoxy-, hydroxyl-tryptamines) catalyzed by *C. roseus* STR (Figure 2). We employed these analogs as a substrate for the entry point of the stemmadenine acetate and alstonine pathways in *N. benthamiana*. We cloned three genes, CrSGD, CrTHAS and CrAS for the production of alstonine and six genes CrSGD, CrGS, CrGO, CrReDOX1, CrReDOX2, and CrSAT for the production of stemmadenine acetate in a binary vector. Each gene was transformed into an individual *A. tumefaciens* GV3101 strain, and then the mixture of strains was infiltrated in our plant heterologous host for the transient expression. These experiments demonstrate that it is possible to reconstitute synthetic pathways for the production of new-to-nature alkaloids by infiltrating a non-natural substrate while co-expressing the pathway genes in *N. benthamiana.*

When precursor-directed biosynthesis was used with *C. roseus* hairy root culture, milligram quantities of selected fluorinated and methylated alkaloids were collected (McCoy et al., 2006). ^1^H-NMR analysis confirmed the production of 5-fluoro-ajmalicine (stereoisomer of tetrahydroalstonine), 5-fluoro, 6-fluoro, and 7-methyl-serpentine (stereoisomer of alstonine) and 6-fluoro and 7-methyl-akuammacine. We made no attempt to heterologously produce ajmalicine and serpentine in this study because the *C. roseus* biosynthetic enzyme that yields these specific stereoisomers has not yet been identified; therefore, in this study we focused on the alternative stereoisomers tetrahydroalstonine and alstonine, for which selective biosynthetic enzymes have been identified. The presence of akuammicine analogs confirmed the stringent substrate specificity of downstream stemmadenine biosynthetic enzymes. Though much lower amounts of alkaloid analogs were observed in this study compared to the 6 alkaloids identified in McCoy et al. (2006) directed biosynthesis in *C. roseus* seedlings and hairy roots yields a mixture of products, while the heterologous reconstitution in *N. benthamiana* allows us to obtain much more defined profiles of fluorinated and chlorinated alstonine analogs, with no contaminating “natural” alkaloid. Notably, we obtained lower amounts of natural alstonine compared to its analogs. The reason for this surprising result is not known, but we hypothesize that natural strictosidine could be more readily transported to other parts of the plant compared to the strictosidine analogs, which would decrease the availability of substrate for conversion to alstonine.

Further studies are required to improve the yields of alkaloid analogs using this system. This is a challenging system in that a number of unstable biosynthetic intermediates (e.g., strictosidine aglycone) comprise this pathway. We envision that engineering the biosynthetic enzymes may improve turnover of the halogenated, methylated and methoxylated substrates. Additionally, optimization of the protein production levels of these complex pathways may also improve the yields of the downstream products. Finally, changing the localization of these enzymes may also improve titers. Nevertheless, with this study we clearly demonstrate that *N. benthamiana* can be used as a platform to produce new-to-nature compounds by combining heterologous transient gene expression with infiltration of an unnatural precursor.

## Data Availability Statement

The datasets presented in this study can be found in online repositories. The names of the repository/repositories and accession number(s) can be found in the article/Supplementary Material.

## Author Contributions

MB and LC performed and designed the experiments. DG performed molecular cloning. AL purified the strictosidine analogs. SO’C assisted with experimental design and managed the project. MB, LC, and SO’C wrote the manuscript. All authors contributed to the article and approved the submitted version.

## Conflict of Interest

The authors declare that the research was conducted in the absence of any commercial or financial relationships that could be construed as a potential conflict of interest.

## Publisher’s Note

All claims expressed in this article are solely those of the authors and do not necessarily represent those of their affiliated organizations, or those of the publisher, the editors and the reviewers. Any product that may be evaluated in this article, or claim that may be made by its manufacturer, is not guaranteed or endorsed by the publisher.

## Footnotes

1
https://www.twistbioscience.com/
