# Supplementary material for: Directed Biosynthesis of New to Nature Alkaloids in a Heterologous Nicotiana benthamiana Expression Host
Source: Front Plant Sci. 2022 Jun 22;13:919443. doi: 10.3389/fpls.2022.919443 (PMC9257203; doi:10.3389/fpls.2022.919443)
Supplement: Supplementary file 1 [file Data_Sheet_1.pdf]

Supplementary Material

Directed biosynthesis of new to nature alkaloids in a heterologous *Nicotiana benthamiana* expression host

Marianna Boccia[1], Dagny Grzech[1], Adriana Lopes[2], Sarah E. O'Connor[1*], Lorenzo Caputi[1*]

[1] Max-Planck Institute for Chemical Ecology, Department of Natural Product Biosynthesis; Hans-Knoll Strasse 8, 07745, Jena, Germany.

[2]Univ Ribeirao Preto UNAERP, Unidade Biotecnol, Av Costabile Romano 2201, BR-14096900 Ribeirao Preto, SP - Brazil

*** Correspondence:**
Corresponding Author
lcaputi@ice.mpg.de

oconnor@ice.mpg.de

Figure S1. Vinblastine biosynthetic pathway starting from the precursor strictosidine.

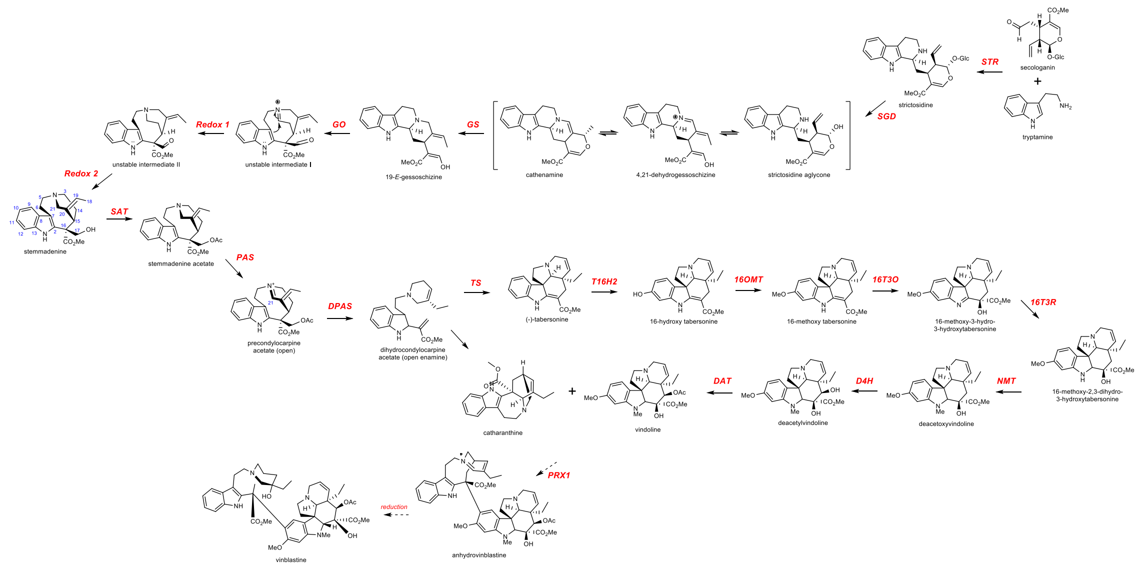

Figure S2. UHPLC-MS chromatograms in MRM mode of 4-fluoro-alstonine produced in *N. benthamiana* leaves produced by co-infiltrating the genes CrSGD, CrTHAS, CrAS. Panel A: MRM chromatogram of alstonine, produced by co-infiltrating with strictosidine. Panel B: MRM chromatogram of 4-fluoro-alstonine produced by co-infiltrating with 4-fluoro-strictosidine. Panel C: MRM chromatogram of the empty vector negative control co-infiltrated with 4-fluoro-strictosidine. Panel D: Fragmentation spectrum of 4-fluoro-alstonine product. 4-fluoro-alstonine=$C_{21}H_{20}O_2N_2F$, Observed *m/z*=367.1444, Theoretical *m/z*=367.1452, Δppm=-2.17. Horizontal axis for panels A, B and C represents the retention time in minutes.

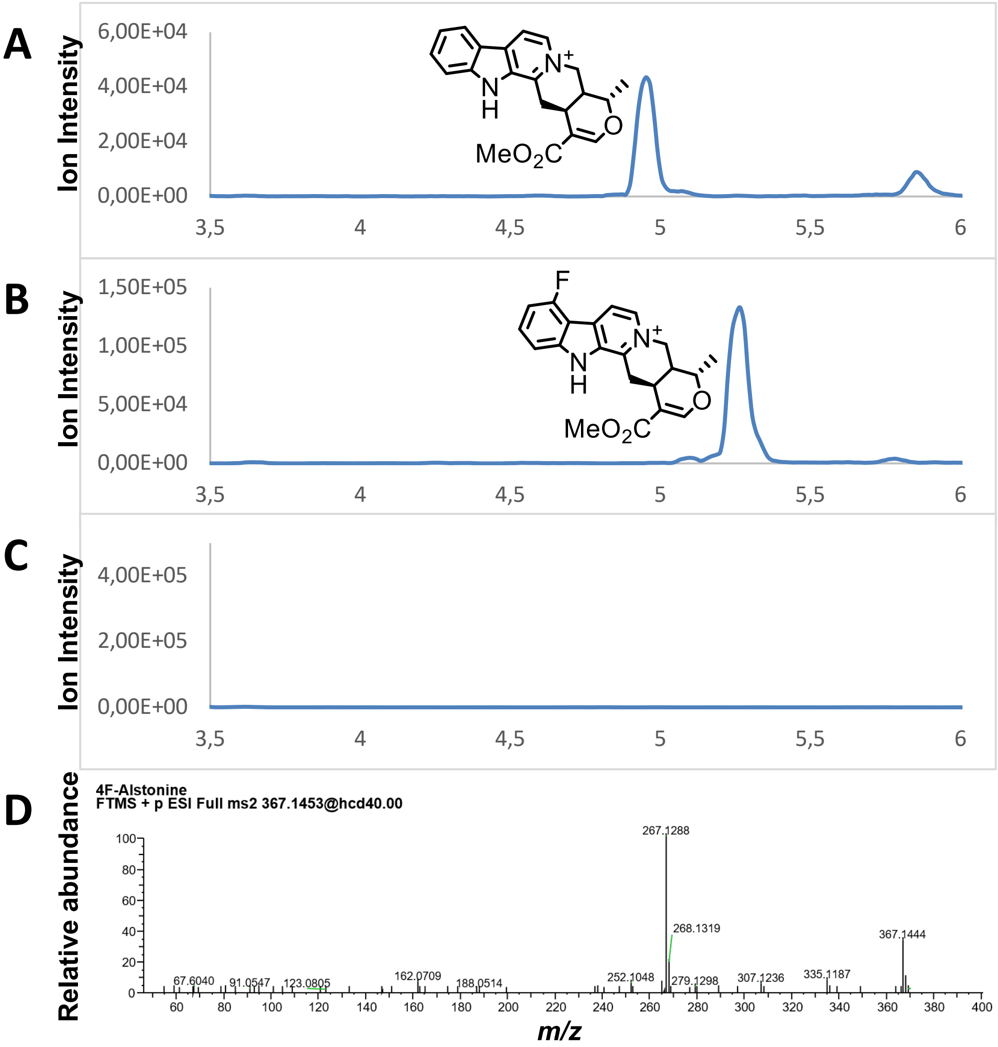

Figure S3. UHPLC-MS chromatograms in MRM mode of 5-fluoro-alstonine produced in *N. benthamiana* leaves produced by co-infiltrating the genes CrSGD, CrTHAS, CrAS. Panel A: MRM chromatogram of alstonine, produced by co-infiltrating with strictosidine. Panel B: MRM chromatogram of 5-fluoro-alstonine produced by co-infiltrating with 5-fluoro-strictosidine. Panel C: MRM chromatogram of the empty vector negative control co-infiltrated with 5-fluoro-strictosidine. Panel D: Fragmentation spectrum of 5-fluoro-alstonine product. 5-fluoro-alstonine=$C_{21}H_{20}O_2N_2F$, Observed *m/z*=367.1452, Theoretical *m/z*=367.1452, Δppm=-0.12. Horizontal axis for panels A, B and C represents the retention time in minutes.

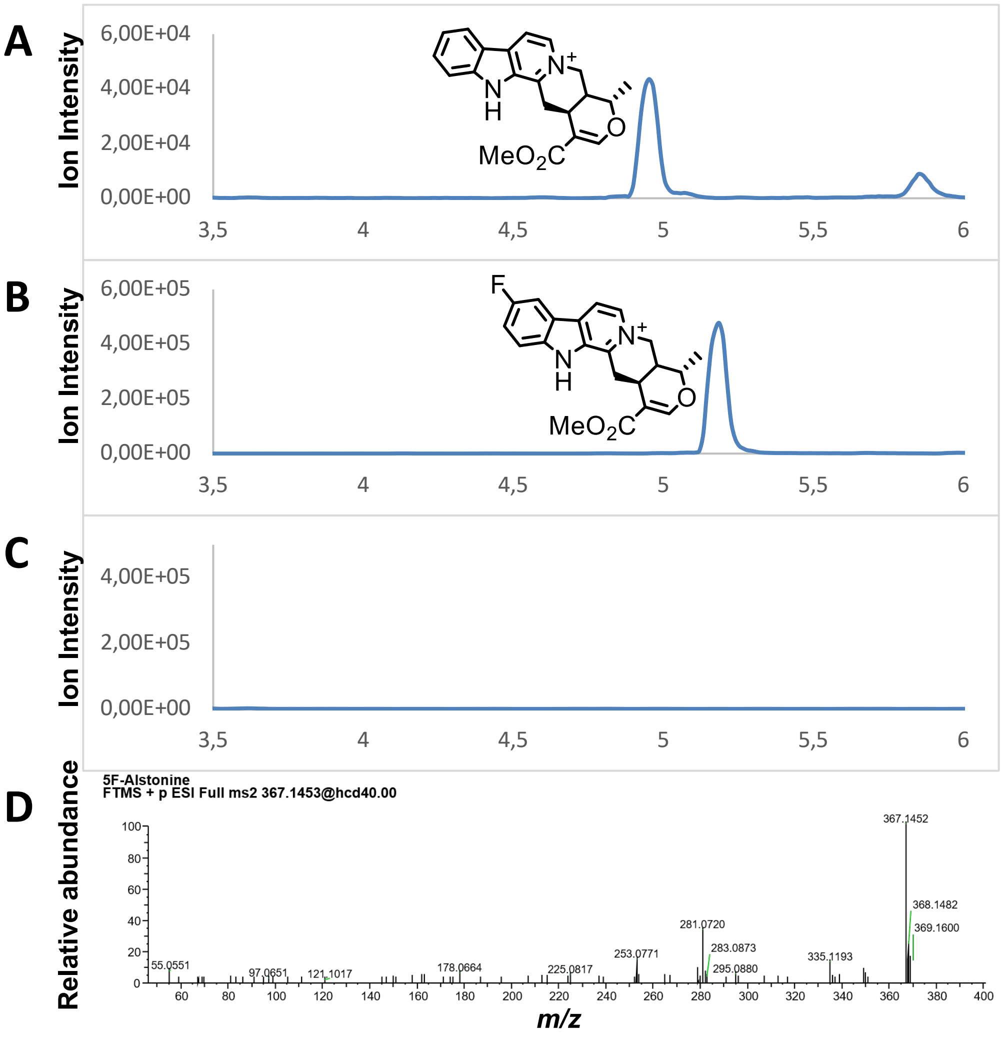

Figure S4. UHPLC-MS chromatograms in MRM mode of 6-fluoro-alstonine produced in *N. benthamiana* leaves produced by co-infiltrating the genes CrSGD, CrTHAS, CrAS. Panel A: MRM chromatogram of alstonine, produced by co-infiltrating with strictosidine. Panel B: MRM chromatogram of 6-fluoro-alstonine produced by co-infiltrating with 6-fluoro-strictosidine. Panel C: MRM chromatogram of the empty vector negative control co-infiltrated with 6-fluoro-strictosidine. Panel D: Fragmentation spectrum of 6-fluoro-alstonine product. 6-fluoro-alstonine=$C_{21}H_{20}O_2N_2F$, Observed *m/z*=367.1449, Theoretical *m/z*=367.1452, Δppm=-0.87. Horizontal axis for panels A, B and C represents the retention time in minutes.

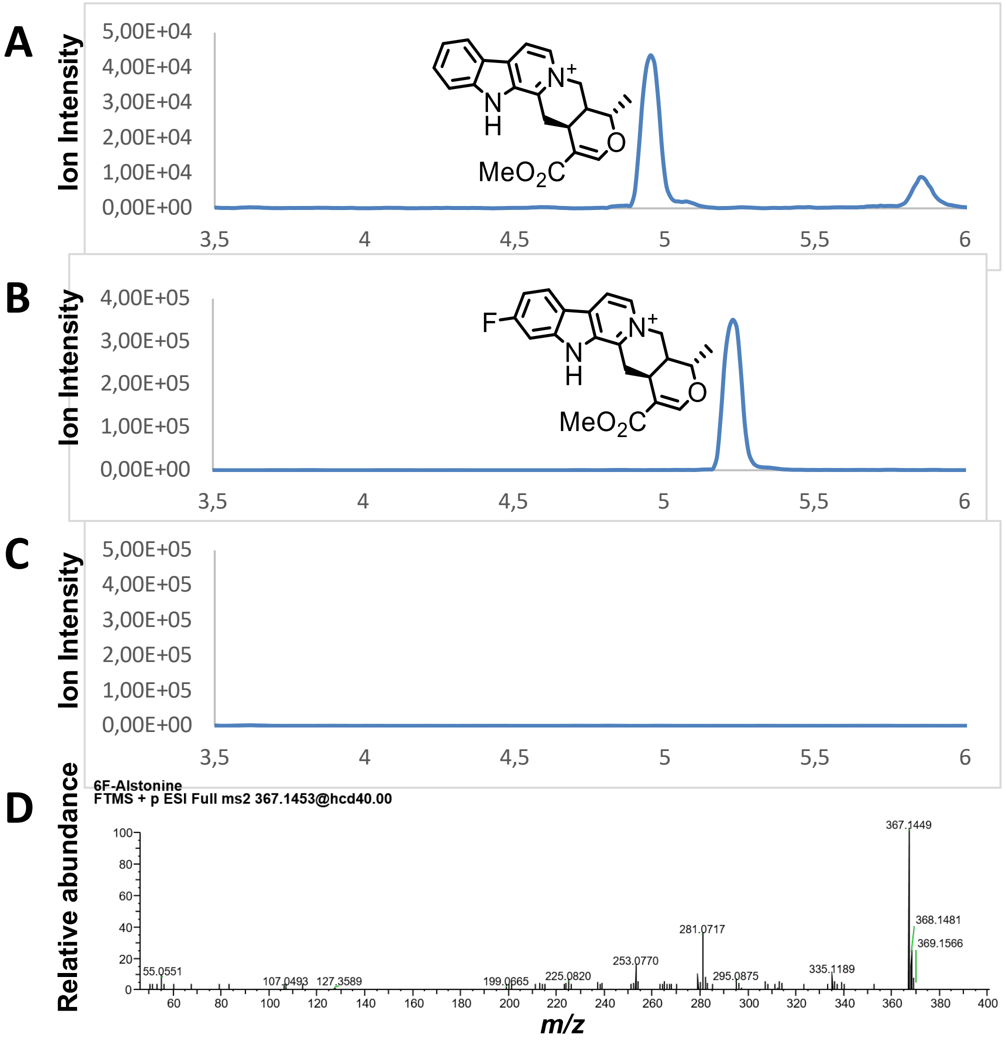

Figure S5. UHPLC-MS chromatograms in MRM mode of 7-fluoro-alstonine produced in *N. benthamiana* leaves produced by co-infiltrating the genes CrSGD, CrTHAS, CrAS. Panel A: MRM chromatogram of alstonine, produced by co-infiltrating with strictosidine. Panel B: MRM chromatogram of 7-fluoro-alstonine produced by co-infiltrating with 7-fluoro-strictosidine. Panel C: MRM chromatogram of the empty vector negative control co-infiltrated with 7-fluoro-strictosidine. Panel D: Fragmentation spectrum of 7-fluoro-alstonine product. 7-fluoro-alstonine=$C_{21}H_{20}O_2N_2F$, Observed *m/z*=367.1451, Theoretical *m/z*=367.1452, Δppm=0.27. Horizontal axis for panels A, B and C represents the retention time in minutes.

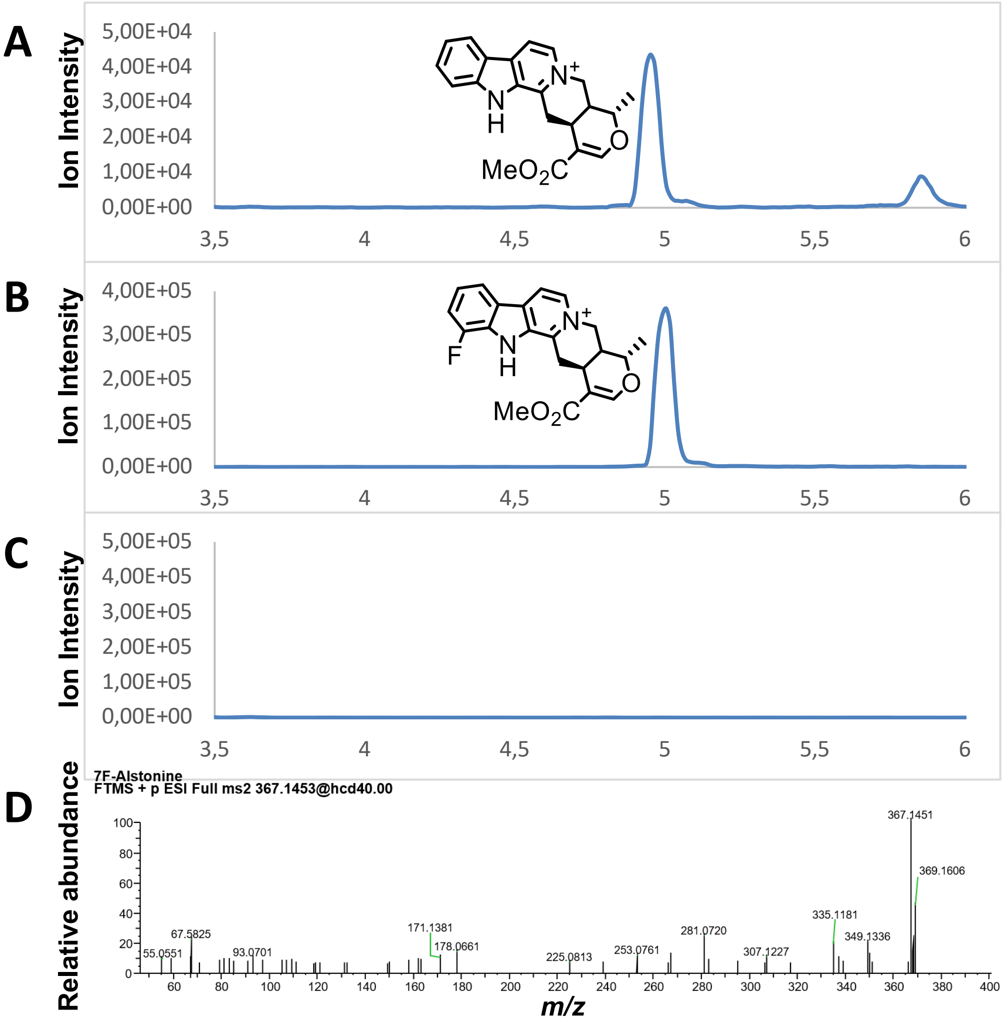

Figure S6. UHPLC-MS chromatograms in MRM mode of 7-chloro-alstonine produced in *N. benthamiana* leaves produced by co-infiltrating the genes CrSGD, CrTHAS, CrAS. Panel A: MRM chromatogram of alstonine, produced by co-infiltrating with strictosidine. Panel B: MRM chromatogram of 7-chloro-alstonine produced by co-infiltrating with 7-chloro-strictosidine. Panel C: MRM chromatogram of the empty vector negative control co-infiltrated with 7-chloro-strictosidine. Panel D: Fragmentation spectrum of 7-chloro-alstonine product. 7-chloro-alstonine=$C_{21}H_{20}O_2N_2Cl$, Observed *m/z*=383.1153, Theoretical *m/z*=383.1157, Δppm=-1.05. Horizontal axis for panels A, B and C represents the retention time in minutes.

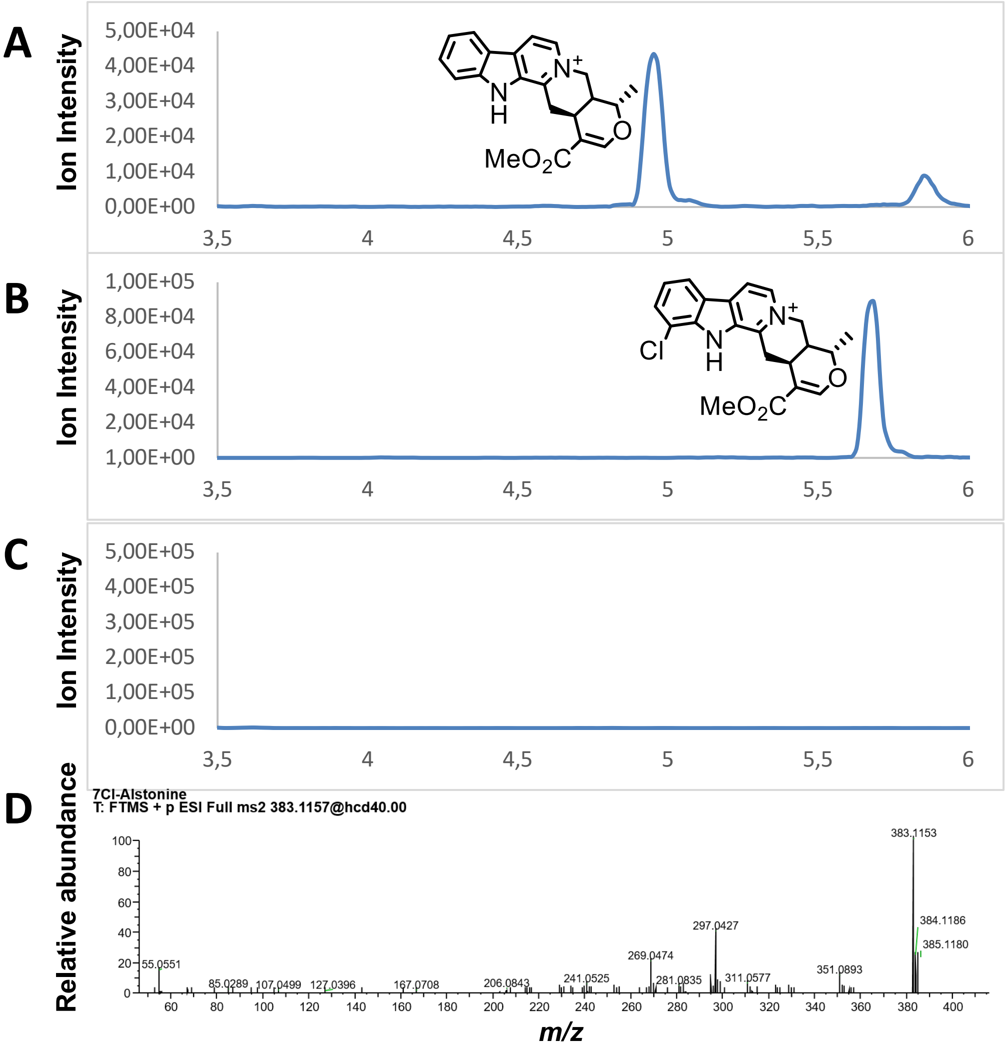

Figure S7. Comparison of the fragmentation spectra of serpentine standard (panel A) and alstonine produced in *N. benthamiana* (panel B) by co-infiltrating the genes CrSGD, CrTHAS, CrAS. Serpentine=$C_{21}H_{21}O_3N_2$, Observed *m/z*=349.1547, Theoretical *m/z*=349.1546, Δppm=0.07. Alstonine=$C_{21}H_{21}O_3N_2$, Observed *m/z*=349.1544, Theoretical *m/z*=349.1546, Δppm=-0.89. The data are in agreement with what reported in the literature (Kumar S., Singh A. Bajpai V., Srivastava M., Singh B.P. and Kumar B., Structural characterization of monoterpene indole alkaloids in ethanolic extracts of Rauwolfia species by liquid chromatography with quadrupole time-of-flight mass pectrometry, *J. Pharm. Anal.*, 2016, 363-373.)

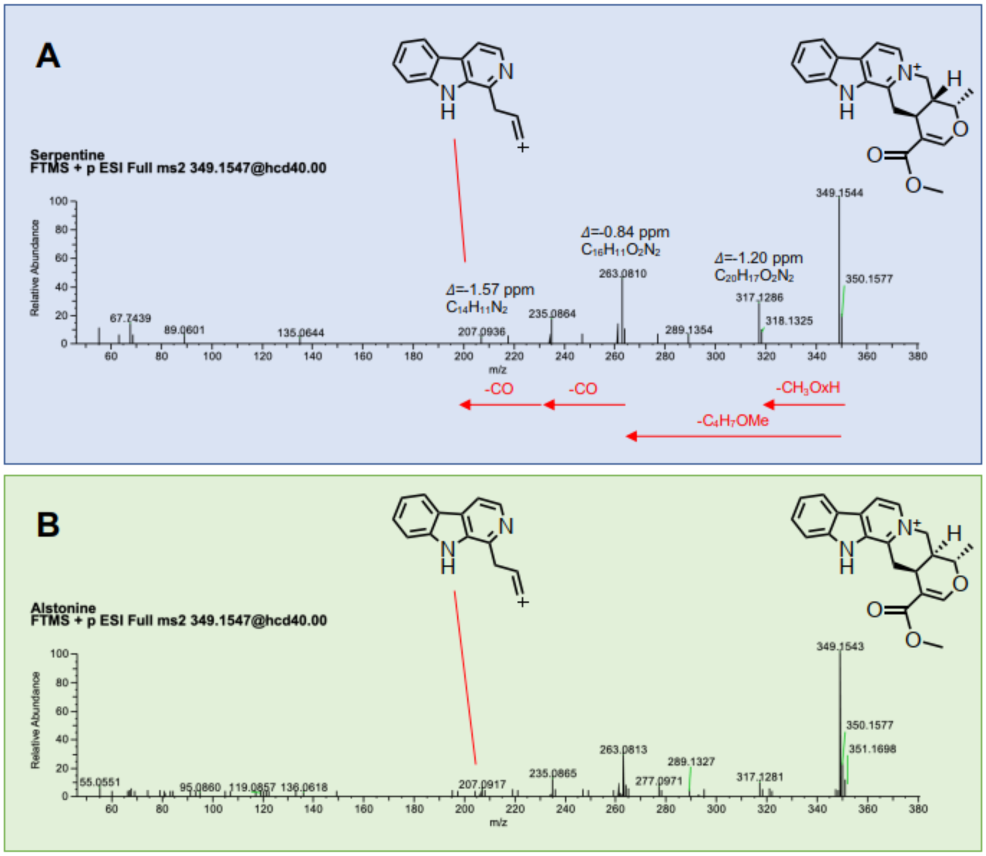

Figure S8. UHPLC-MS chromatograms in MRM mode of methyl and methoxy-tetrahydroalstonine produced in *N. benthamiana* leaves produced by co-infiltrating the genes CrSGD, CrTHAS, CrAS. Panel A: MRM chromatogram of tetrahydroalstonine, produced by co-infiltrating with strictosidine. Panel B: MRM chromatogram of 6-methyl-tetrahydroalstonine produced by co-infiltrating with 6-methyl-strictosidine. Panel C: MRM chromatogram of 5-methoxy-tetrahydroalstonine produced by co-infiltrating with 5-methoxy-strictosidine. Panel D: MRM chromatogram of the empty vector negative control co-infiltrated with 5-methoxy- or 6-methyl-strictosidine. *unknown compound. Horizontal axis represents the retention time in minutes.

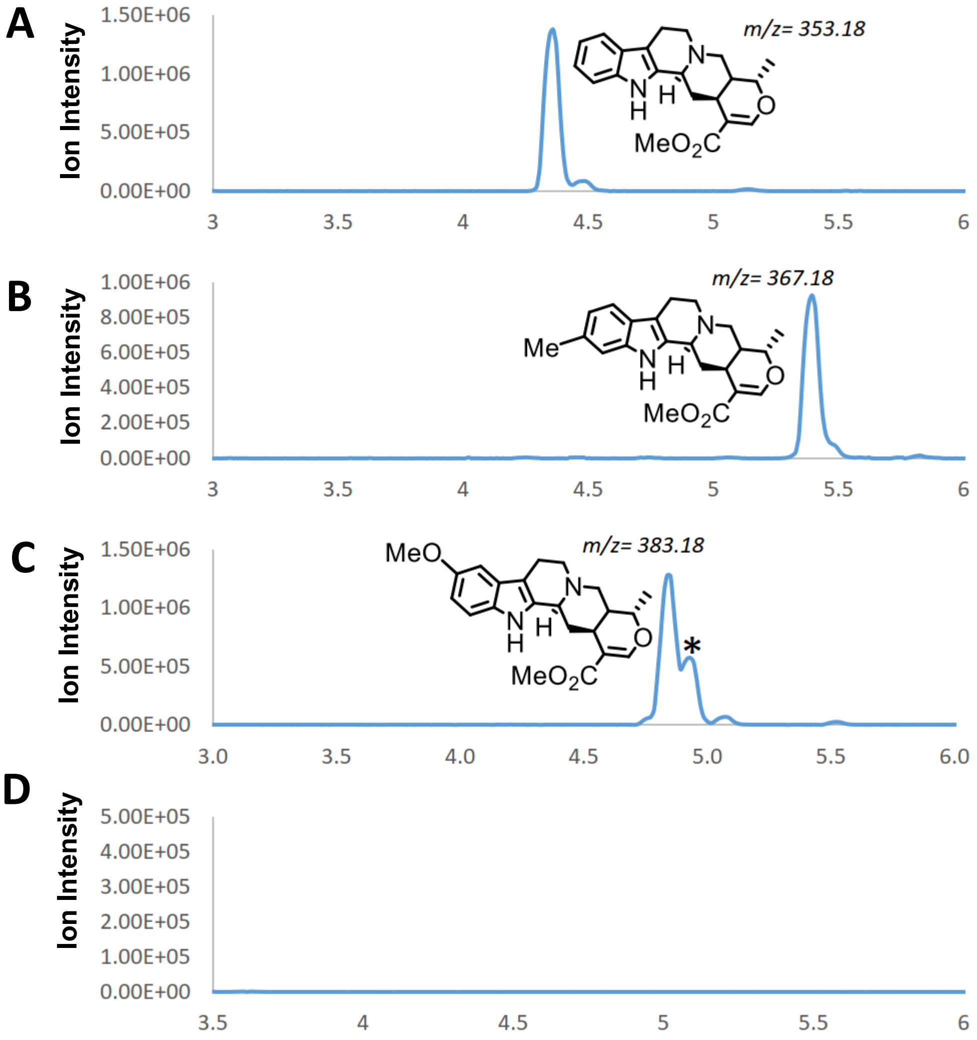

Figure S9. Fragmentation spectrum of stemmadenine acetate standard. Molecular Formula= $C_{23}H_{28}O_4N_2$, $[M+H]^+$ Observed *m/z*=397.2115, Theoretical *m/z*=397.2121, Δppm=-1.60.

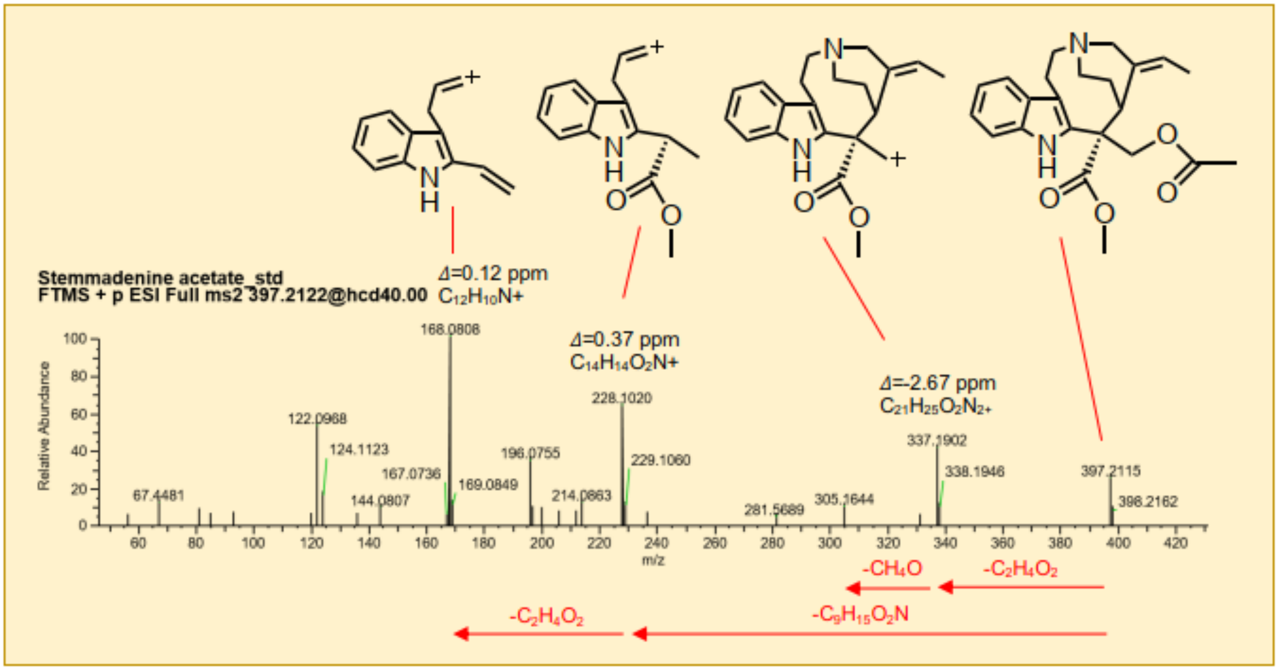

Figure S10. UHPLC-MS chromatograms in MRM mode of 4-fluoro-stemmadenine acetate produced in *N. benthamiana* leaves by co-infiltrating the genes CrSGD, CrGS, CrGO, CrRedOx1, CrRedOx2, CrSAT. Panel A: MRM chromatogram of stemmadenine acetate, produced by co-infiltrating with strictosidine. Panel B: MRM chromatogram of 4-fluoro-stemmadenine acetate, produced by co-infiltrating with 4-fluoro-strictosidine. Panel C: MRM chromatogram of the empty vector negative control co-infiltrated with 4-fluoro-strictosidine. Panel D: Fragmentation spectrum of 4-fluoro-stemmadenine acetate product. 4-fluoro-stemmadenine acetate=$C_{23}H_{28}O_4N_2F$, Observed *m/z*=415.2018, Theoretical *m/z*=415.2027, Δppm=-2.29. Horizontal axis for panels A, B and C represents the retention time in minutes.

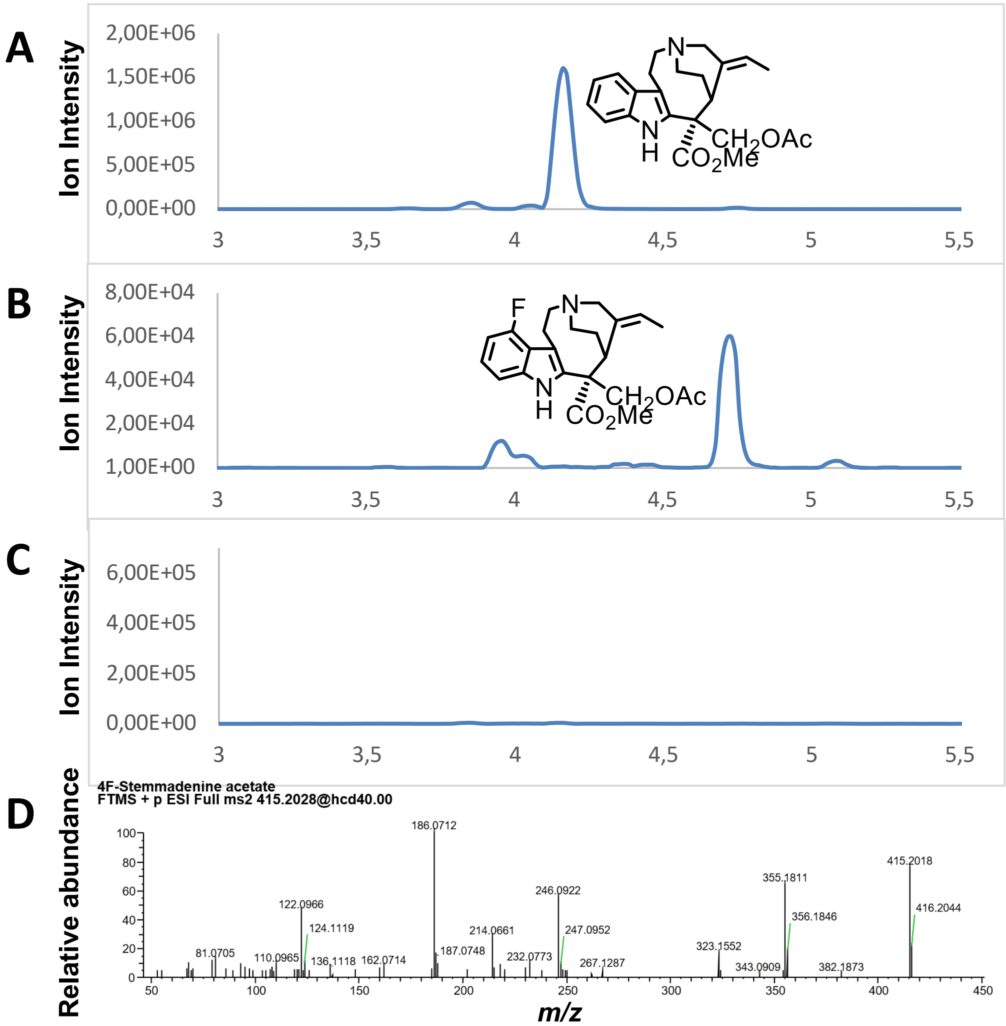

Figure S11. UHPLC-MS chromatograms in MRM mode of 5-fluoro-stemmadenine acetate produced in *N. benthamiana* leaves by co-infiltrating the genes CrSGD, CrGS, CrGO, CrRedOx1, CrRedOx2, CrSAT. Panel A: MRM chromatogram of stemmadenine acetate, produced by co-infiltrating with strictosidine. Panel B: MRM chromatogram of 5-fluoro-stemmadenine acetate, produced by co-infiltrating with 5-fluoro-strictosidine. Panel C: MRM chromatogram of the empty vector negative control co-infiltrated with 5-fluoro-strictosidine. Panel D: Fragmentation spectrum of 5-fluoro-stemmadenine acetate product. 5-fluoro-stemmadenine acetate=$C_{23}H_{28}O_4N_2F$, Observed *m/z*=415.2023, Theoretical *m/z*=415.2027, Δppm=0.96. Horizontal axis for panels A, B and C represents the retention time in minutes.

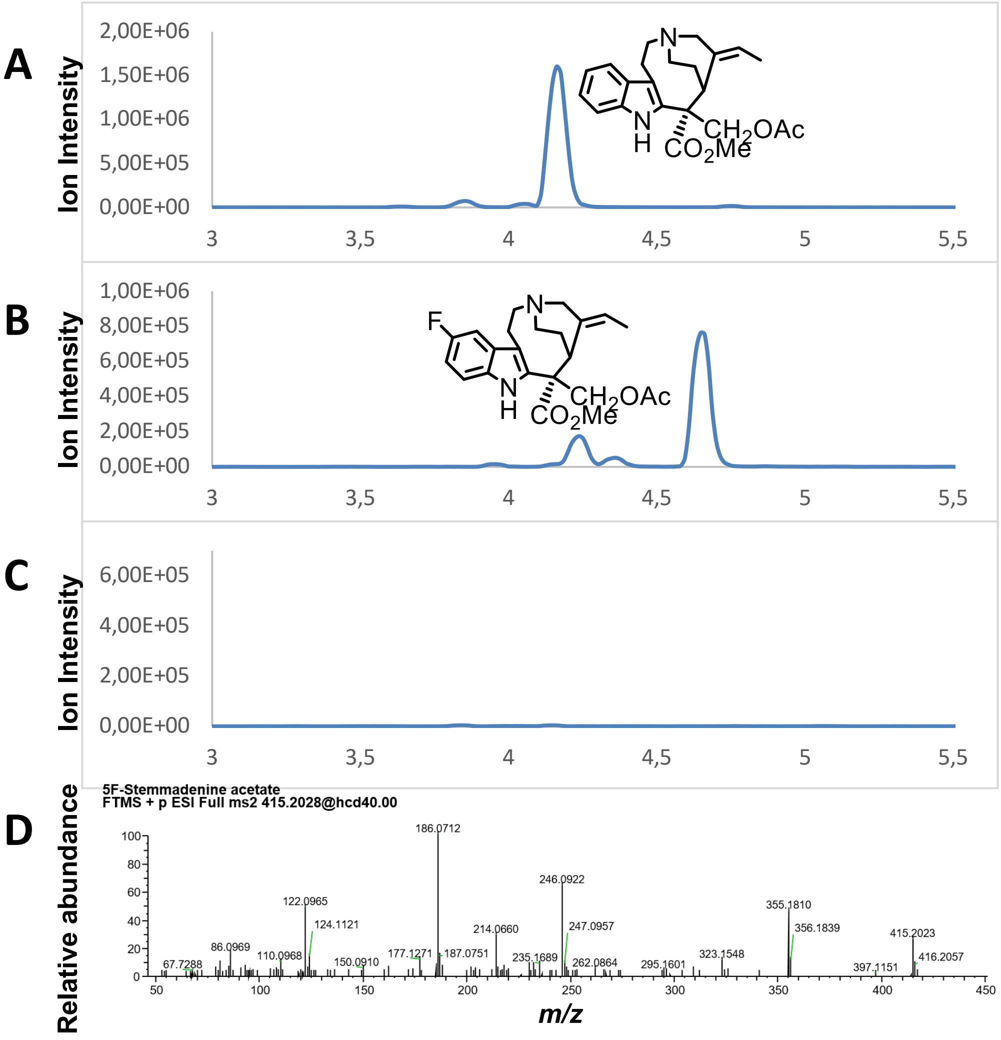

Figure S12. UHPLC-MS chromatograms in MRM mode of 6-fluoro-stemmadenine acetate produced in *N. benthamiana* leaves by co-infiltrating the genes CrSGD, CrGS, CrGO, CrRedOx1, CrRedOx2, CrSAT. Top panel: MRM chromatogram of stemmadenine acetate, produced by co-infiltrating with strictosidine. Middle panel: MRM chromatogram of 6-fluoro-stemmadenine acetate, produced by co-infiltrating with 6-fluoro-strictosidine. Bottom panel: MRM chromatogram of the empty vector negative control co-infiltrated with 6-fluoro-strictosidine. Panel D: Fragmentation spectrum of 6-fluoro-stemmadenine acetate product. 6-fluoro-stemmadenine acetate=$C_{23}H_{28}O_4N_2F$, Observed *m/z*=415.2018, Theoretical *m/z*=415.2027, Δppm=-1.62. Horizontal axis for panels A, B and C represents the retention time in minutes.

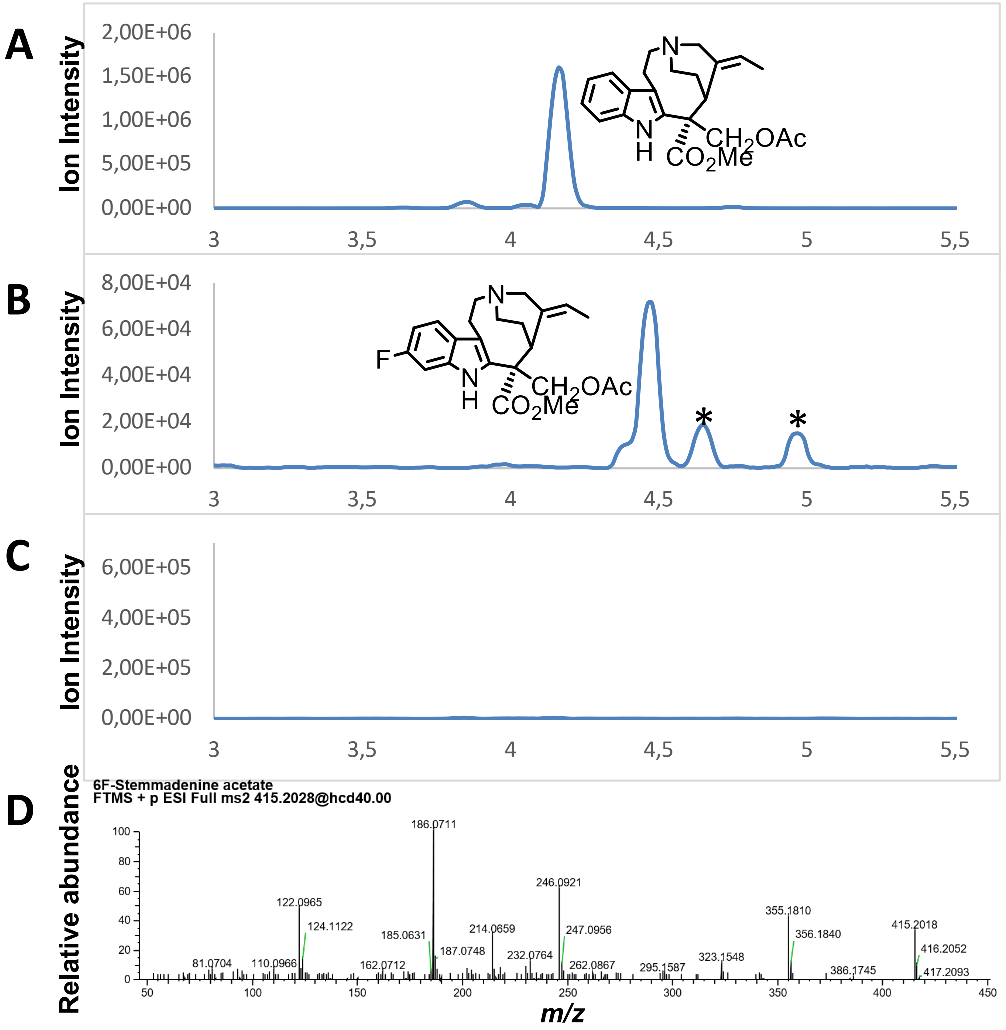

Figure S13. UHPLC-MS chromatograms in MRM mode of 7-fluoro-stemmadenine acetate produced in *N. benthamiana* leaves by co-infiltrating the genes CrSGD, CrGS, CrGO, CrRedOx1, CrRedOx2, CrSAT. Panel A: MRM chromatogram of stemmadenine acetate, produced by co-infiltrating with strictosidine. Panel B: MRM chromatogram of 7-fluoro-stemmadenine acetate, produced by co-infiltrating with 7-fluoro-strictosidine. Panel C: MRM chromatogram of the empty vector negative control co-infiltrated with 7-fluoro-strictosidine. Horizontal axis for panels A, B and C represents the retention time in minutes.

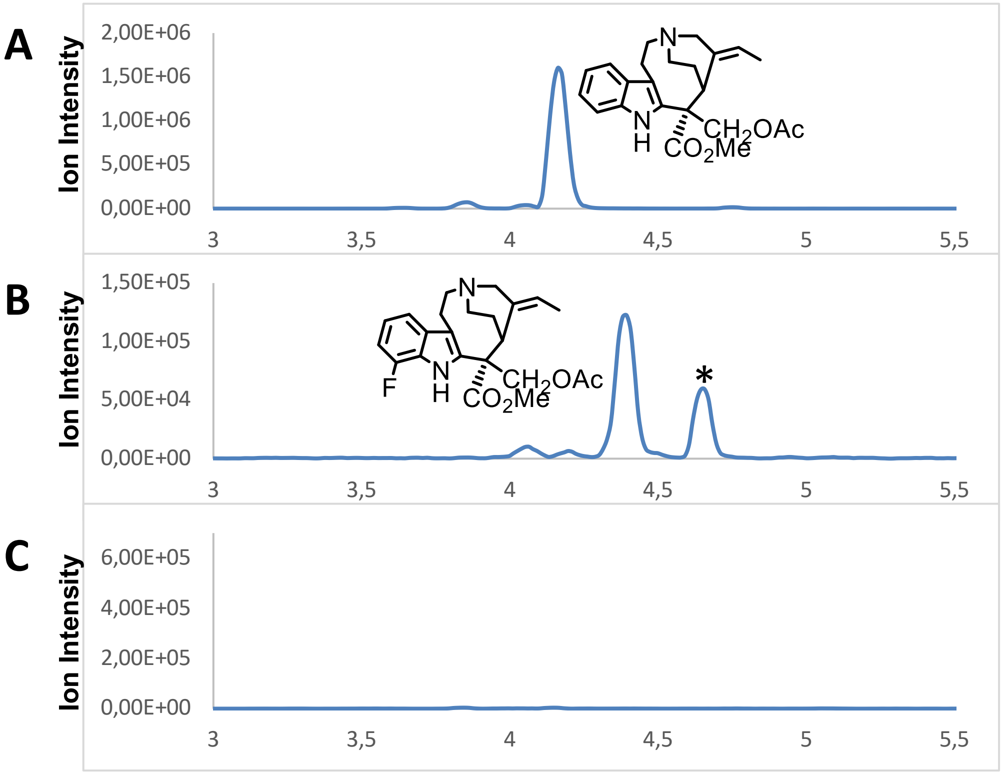

Figure S14. UHPLC-MS chromatograms in MRM mode of the shunt product fluoro-akuammicine produced in *N. benthamiana* leaves produced by co-infiltrating the genes CrSGD, CrGS, CrGO, CrRedOx1, CrRedOx2, CrSAT. Panel A: MRM chromatogram of akuammicine, produced by co-infiltrating with strictosidine. Panels B and C: MRM chromatogram of 4, 5, 6, and 7-fluoro-akuammicine, produced by co-infiltrating with 4, 5, 6, 7-fluoro-strictosidine, respectively. Panel D: MRM chromatogram of the empty vector negative control co-infiltrated with 5-fluoro-strictosidine. Horizontal axis represents the retention time in minutes.

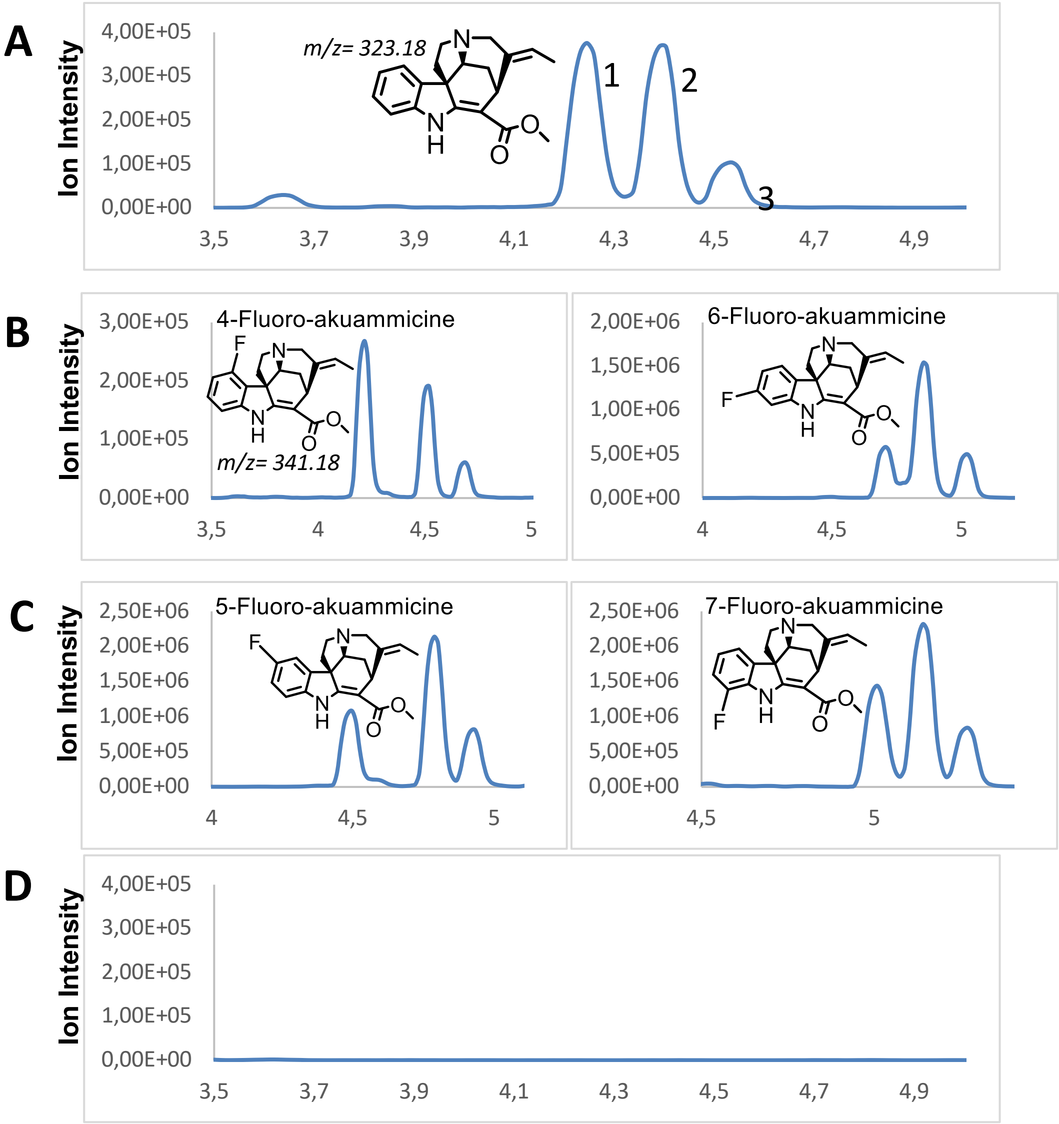

Figure S15. UHPLC-MS chromatograms in MRM mode of fluoro-precondylocarpine acetate produced in *N. benthamiana* leaves produced by co-infiltrating the genes CrSGD, CrGS, CrGO, CrRedOx1, CrRedOx2, CrSAT. Panel A: MRM chromatogram of precondylocarpine acetate, produced by co-infiltrating with strictosidine. Panels B and C: MRM chromatogram of 4, 5, 6, and 7-fluoro- precondylocarpine acetate, produced by co-infiltrating with 4, 5, 6, 7-fluoro-strictosidine, respectively. Panel D: MRM chromatogram of the empty vector negative control co-infiltrated with 5-fluoro-strictosidine. Horizontal axis represents the retention time in minutes.

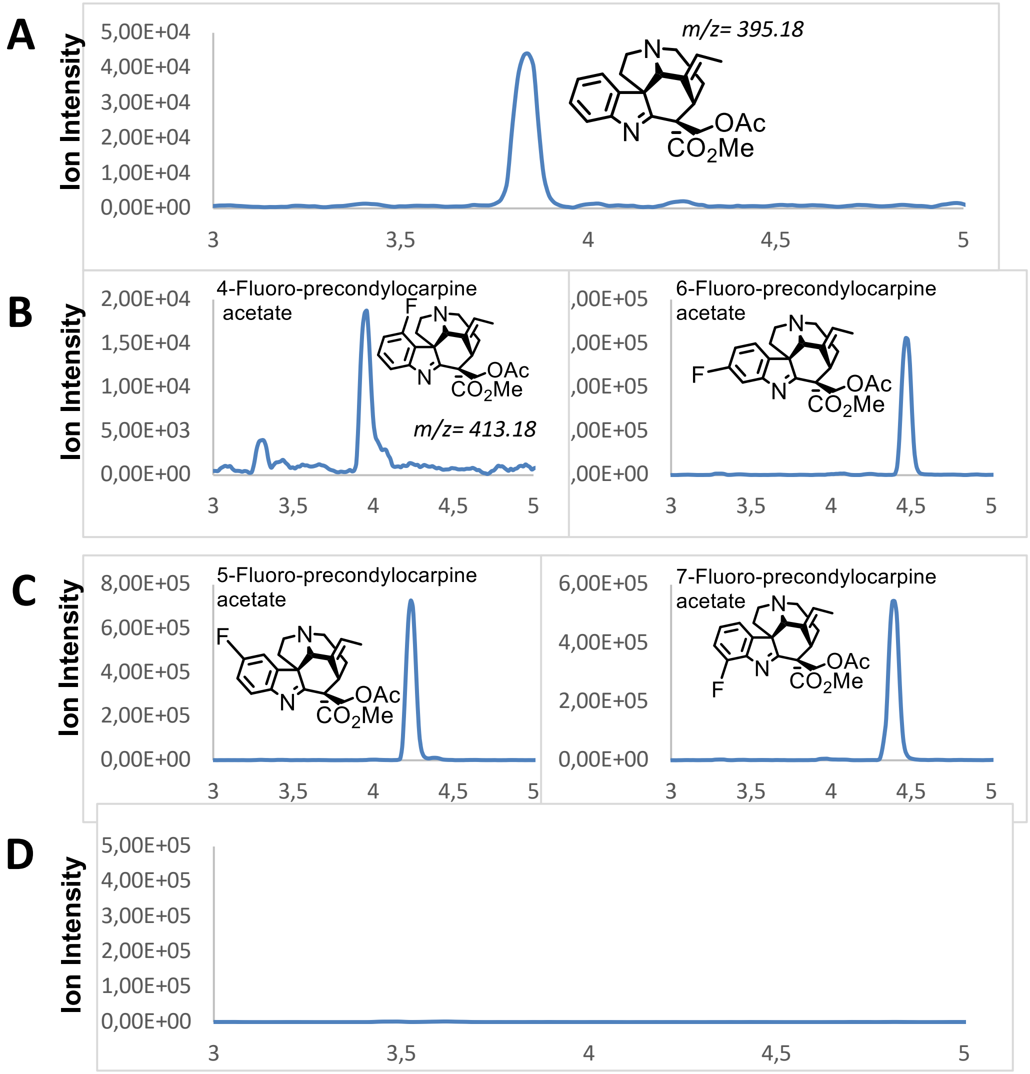

Figure S16. UHPLC-MS chromatograms in MRM mode of the shunt product 6-methyl-akuammicine produced in *N. benthamiana* leaves produced by co-infiltrating the genes CrSGD, CrGS, CrGO, CrRedOx1, CrRedOx2, CrSAT. Panel A: MRM chromatogram of akuammicine, produced by co-infiltrating with strictosidine. Panel B: MRM chromatogram of 6-methyl-akuammicine, produced by co-infiltrating with 6-methyl-strictosidine, respectively. Panel 3: MRM chromatogram of 7-methyl-akuammicine, produced by co-infiltrating with 7-methyl-strictosidine, respectively. Panel D: MRM chromatogram of the empty vector negative control co-infiltrated with 6-methyl-strictosidine. #1; #2; #3 akuammicine isomers. Horizontal axis represents the retention time in minutes.

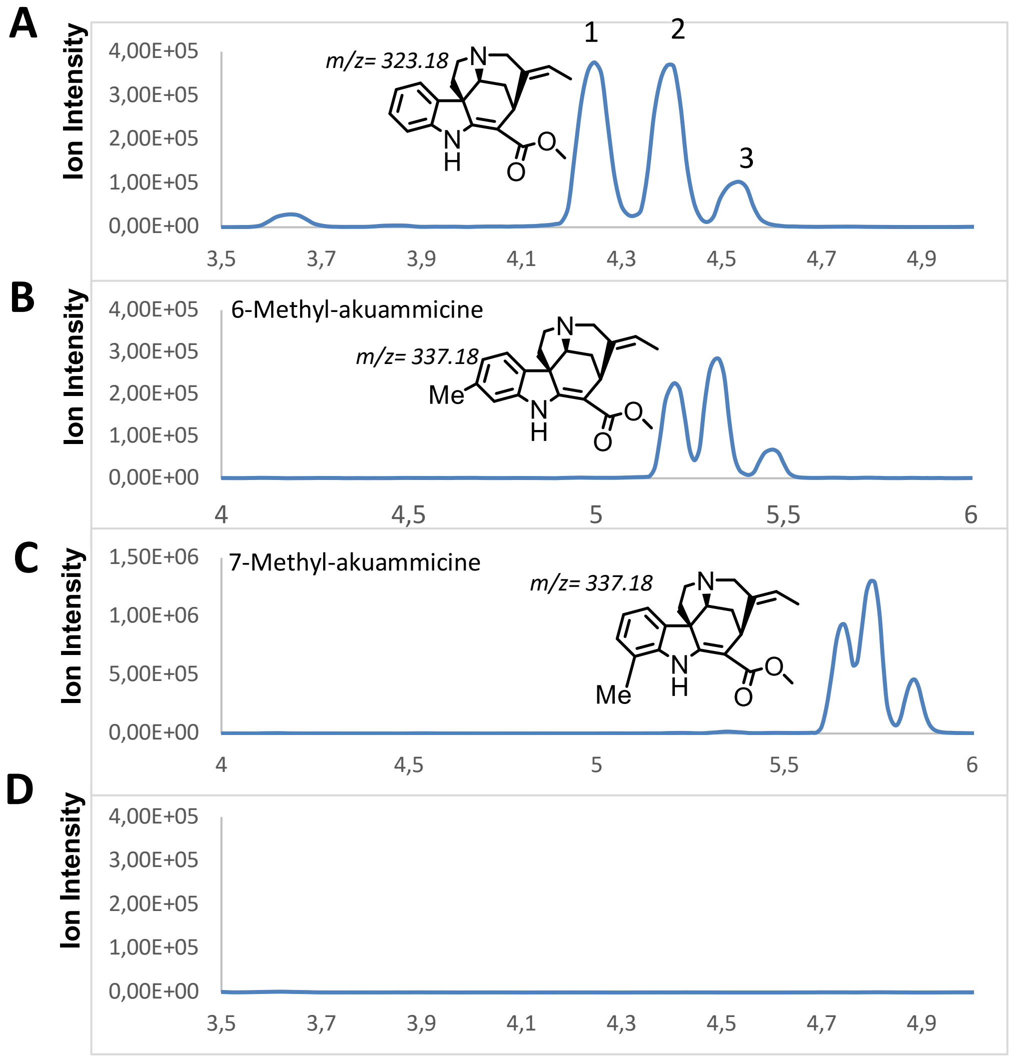

Figure S17. UHPLC-MS chromatograms in MRM mode of alkaloids produced in *N. benthamiana* leaves produced by co-infiltrating the genes CrSGD, CrGS, CrGO, CrRedOx1, CrRedOx2, CrSAT and chloro-strictosidine as substrate. Panel A: MRM chromatogram of stemmadenine acetate (left) and 6-Chloro-stemmadenine acetate (right), produced by co-infiltrating with strictosidine and 6-chloro-strictosidine, respectively. Panel B: MRM chromatogram of akuammicine and 6-chloro-akuammicine, produced by co-infiltrating with strictosidine and 6-chloro-strictosidine, respectively. Panel C: MRM chromatogram of MRM trace of precondylocarpine acetate (left) and 6-chloro-precondylocarpine acetate (right), produced by co-infiltrating with strictosidine and 6-chloro-strictosidine, respectively. Horizontal axis represents the retention time in minutes.

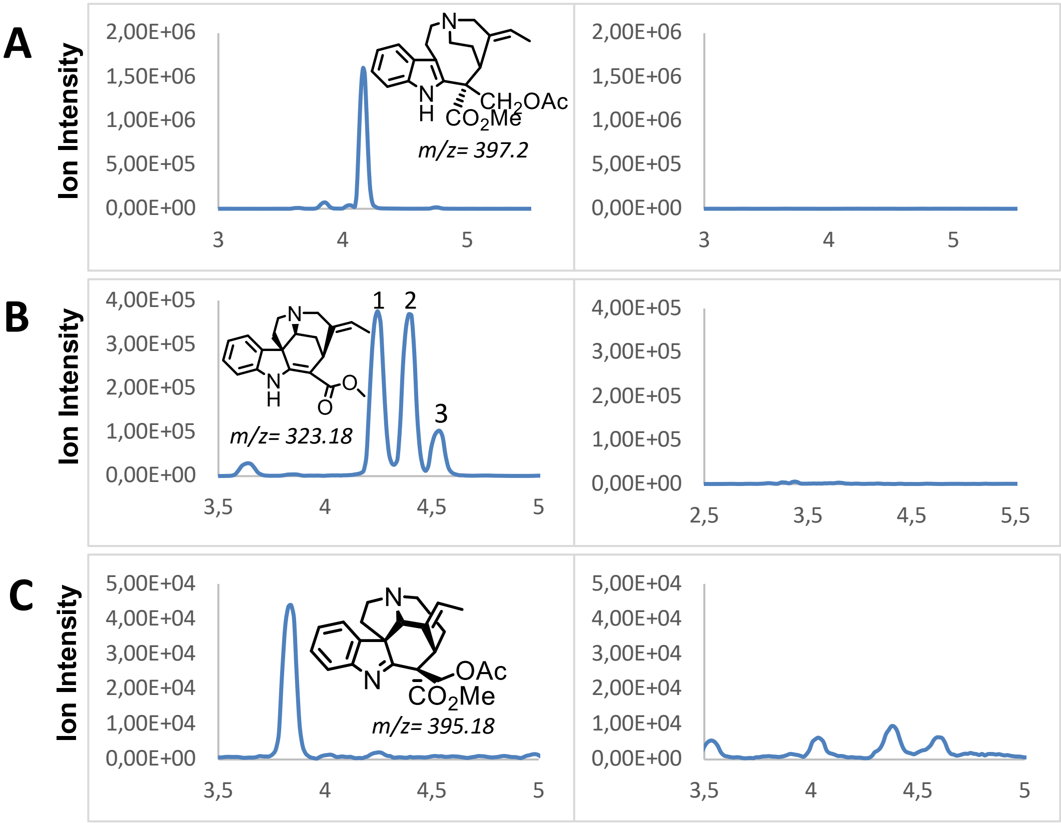

Table S1. Tryptamine analogs

Compound	*Company*
4-Fluoro tryptamine	Sigma Aldrich
5-Fluoro tryptamine	Sigma Aldrich
6-Fluoro tryptamine	Sigma Aldrich
7-Fluoro tryptamine	Sigma Aldrich
4-Methoxy tryptamine	Sigma Aldrich
5-Methoxy tryptamine	Sigma Aldrich
6-Methoxy tryptamine	Sigma Aldrich
7-Methoxy tryptamine	Sigma Aldrich
5-Chloro tryptamine	Alfa Aesar
6-Chloro tryptamine	Cayman chemical
7-Chloro tryptamine	Sigma Aldrich
5-Methyl tryptamine	Sigma Aldrich
6-Methyl tryptamine	BIOSYNTH
7-Methyl tryptamine	Sigma Aldrich
6-Hydroxy tryptamine	Sigma Aldrich
7-Bromo tryptamine	Fluorochem
6-Hydroxy tryptamine	TRC

Table S2. Sequences of the genes used in this study. Genbank accession numbers: CrSGD (AF112888), CrGS (MF770507), CrGO (MF770508), CrRedox1 (MF770509), CrRedox2 (MF770510), CrSAT (MF770511), CrTHAS (KM524258.1), CrAS (CYP71AY1), SlUbi10p and SlUbi10t (SOLYC01G096290.2).

CrSGD	AGGTCTCTAATGGGTTCTAAGGATGATCAATCACTTGTCGTGGCTATCAGTCCTGCCG<br>CCGAGCCTAACGGGAACCACTCAGTTCCTATACCGTTTGCATATCCGAGCATACCTAT<br>ACAGCCGCGAAAACATAATAAACCTATAGTACACAGGCGGGACTTTCCTTCTGACTT<br>TATTCTTGGAGCTGGCGGTTCCGCGTACCAATGCGAAGGCGCTTACAACGAGGGAAA<br>TAGAGGACCAAGCATTTGGGACACATTTACTAATAGATACCCTGCAAAGATTGCAGA<br>CGGCAGTAACGGAAACCAGGCAATTAACAGCTATAACTTATACAAAGAGGACATAA<br>AGATAATGAAACAGACTGGGCTTGAGAGCTACCGATTCTCTATCTCTTGGTCCCGAGT<br>TCTGCCTGGTGGAAACTTGAGTGGCGGGGTTAACAAGGACGGAGTGAAATTTTACCA<br>CGATTTCATTGACGAACTACTGGCAAACGGTATTAAGCCTTTCGCCACCTTGTTTCAT<br>TGGGACCTGCCTCAGGCGTTAGAAGATGAATACGGTGGATTTCTCAGCGACCGCATA<br>GTTGAGGACTTCACAGAATACGCTGAGTTCTGTTTCTGGGAGTTTGGCGATAAGGTTA<br>AGTTCTGGACAACATTTAACGAGCCTCACACATACGTCGCTTCAGGGTACGCTACGG<br>GAGAGTTCGCCCCCGGCAGGGGCGGAGCTGACGGAAAAGGTGAGCCAGGAAAGGAG<br>CCTTACATTGCTACCCACAACCTTTTGCTCTCACATAAGGCAGCAGTAGAGGTTTACA<br>GAAAGAACTTCCAAAAGTGCCAGGGCGGAGAGATCGGCATAGTCTTAAACTCTATGT<br>GGATGGAACCCCTTAACGAGACTAAGGAAGATATCGACGCACGCGAGCGTGGACTC<br>GACTTTATGCTTGGTTGGTTTATTGAACCTCTCACTACAGGAGAGTATCCTAAGAGTA<br>TGCGGGCACTCGTTGGTAGTCGGTTGCCTGAGTTCTCCACAGAGGTGTCAGAGAAGC<br>TCACTGGCTGTTACGACTTCATTGGTATGAACTACTACACTACAACATACGTAAGCAA<br>CGCCGATAAGATCCCTGACACCCCTGGATATGAAACCGACGCAAGAATCAACAAGA<br>ACATATTCGTGAAGAAGGTAGACGGTAAAGAGGTTAGAATAGGCGAGCCATGTTAC<br>GGTGGTTGGCAACACGTGGTGCCTTCCGGTCTATATAACCTACTTGTGTATACCAAAG<br>AAAAGTATCACGTACCCGTTATATACGTTAGCGAGTGCGGCGTAGTAGAAGAGAACA<br>GGACAAATATTTTGTTGACTGAGGGCAAGACAAATATCCTTTTGACTGAGGCCAGAC<br>ATGACAAGCTTCGAGTGGACTTCTTGCAGTCACACCTAGCCTCAGTTCGGGACGCAA<br>TAGACGACGGAGTCAACGTTAAGGGGTTCTTCGTATGGAGTTTCTTTGATAATTTTGA<br>GTGGAACCTTGGTTACATCTGTAGATACGGTATAATTCACGTAGACTACAAGACATTC<br>CAGCGATACCCTAAAGACTCCGCTATTTGGTATAAGAACTTTATCTCAGAGGGCTTCG<br>TGACTAACACCGCAAAGAAACGTTTTAGGGAAGAGGACAAGCTGGTAGAACTTGTTA<br>AGAAACAGAAGTATTAAGCTTAGAGACCT
CrGS	ATGGCCGGAGAAACAACCAAACTCGACCTTTCAGTGAAGGCTGTGGGATGGGGTGCT<br>GCAGATGCATCTGGTGTCCTTCAGCCCATCAAGTTCTATAGAAGAGTCCCTGGTGAAC<br>GGGATGTGAAGATTAGAGTTTTGTACTCTGGTGTTTGCAATTTCGATATGGAAATGGT<br>CAGAAACAAGTGGGGTTTCACCAGATATCCTTATGTGTTTGGACATGAAACTGCCGG<br>TGAGGTGGTAGAAGTTGGCAGCAAAGTAGAGAAATTCAAGGTTGGAGACAAGGTAG<br>CTGTGGGATGTATGGTCGGATCTTGTGGTCAATGTTATAATTGTCAAAGTGGAATGGA<br>GAATTACTGCCCAGAGCCCAATATGGCTGATGGATCTGTTTACCGTGAGCAAGGGGA<br>ACGATCCTATGGGGGTTGTTCAAATGTGATGGTTGTTGATGAAAAGTTCGTCCTCCGA<br>TGGCCCGAAAACTTGCCTCAAGATAAAGGGGTTGCTCTCCTTTGTGCTGGGGTTGTTG<br>TTTATAGCCCAATGAAACATTTGGGACTCGATAAGCCAGGAAAGCATATTGGGGTTT<br>TCGGGCTGGGAGGTCTTGGTTCTGTTGCTGTTAAGTTTATTAAGGCTTTTGGTGGTAA<br>GGCTACTGTTATTAGTACATCAAGGCGTAAGGAGAAGGAAGCCATTGAAGAACATGG<br>TGCTGATGCTTTTGTTGTCAACACTGACTCTGAGCAATTGAAGGCTCTGGCAGGTACT<br>ATGGATGGTGTTGTGGACACCACCCCAGGTGGCCGCACTCCTATGTCACTTATGCTCA<br>ATTTGCTCAAGTTTGACGGCGCGGTTATGCTCGTAGGTGCACCGGAGTCGCTATTTGA<br>GCTCCCTGCGGCACCTCTCATTATGGGAAGGAAAAAGATAATCGGAAGTTCCACTGG<br>AGGCCTCAAAGAGTACCAAGAAATGCTTGATTTCGCAGCCAAACATAACATTGTATG<br>TGATACTGAAGTTATTGGGATTGACTATCTCAGCACTGCTATGGAACGTATCAAGAAT<br>TTGGATGTCAAGTACCGTTTTGCGATTGACATTGGAAATACATTGAAATTTGAGGAAT<br>AA

CrGO	ATGGAGTTTTCTTTCTCCTCACCAGCTCTCTACATAGTTTATTTCTTGCTTTTCTTTGTG GTAAGGCAATTATTGAAACCCAAAAGTAAGAAAAAATTGCCACCAGGTCCAAGAAC ACTACCCTTAATTGGAAACCTTCATCAACTCTCGGGACCTTTACCTCATCGTACCCTA AAAAATTTGTCCGATAAACATGGTCCTTTGATGCACGTGAAAATGGGCGAACGTTCG GCAATTATAGTATCAGATGCAAGAATGGCAAAAATAGTTCTTCATAATAACGGTTTA GCCGTTGCAGATCGGTCAGTAAATACTGTCGCAAGTATTATGACTTATAATAGTTTGG GTGTTACCTTTGCTCAGTATGGAGATTACTTAACAAAATTACGTCAAATCTATACTTT GGAACTTTTAAGTCAGAAAAAAGTTCGATCTTTCTACAGTTGTTTTGAAGATGAACTC GATACTTTTGTTAAGTCAATTAAGTCTAACGTTGGACAACCTATGGTTTTGTACGAGA AAGCTTCTGCTTATTTGTATGCTACCATTTGTAGAACTATTTTTGGGAGTGTTTGCAAG GAAAAGGAGAAAATGATTAAGATAGTGAAGAAAACGTCGTTACTGTCTGGAACACC ACTTAGATTGGAGGATCTTTTTCCTTCTATGAGCATTTTTTGTAGATTTAGTAAAACTT TGAATCAATTGAGAGGACTTTTGCAAGAAATGGATGATATTTTGGAAGAAATCATAG TTGAAAGGGAAAAGGCTTCTGAAGTCTCAAAAGAGGCAAAGGATGATGAAGATATG CTTAGTGTTCTGTTGAGGCATAAATGGTACAATCCAAGTGGTGCTAAATTCAGAATCA CTAATGCAGACATCAAAGCCATTATCTTTGAGTTGATCTTAGCAGCAACCCTAAGTGT AGCAGATGTTACAGAATGGGCTATGGTTGAAATTCTAAGGGATCCCAAAAGTTTGAA AAAAGTGTACGAAGAAGTACGAGGTATTTGTAAAGAAAAGAAAAGAGTGACAGGAT ACGACGTTGAAAAAATGGAATTTATGCGTCTTTGTGTGAAGGAATCAACAAGAATAC ATCCAGCTGCTCCTCTTTTAGTTCCTAGAGAATGCAGAGAAGATTTTGAAGTAGATGG ATACACAGTTCCAAAAGGGGCTTGGGTTATAACAAATTGCTGGGCTGTTCAAATGGA TCCTACAGTATGGCCCGAACCAGAGAAGTTTGATCCAGAAAGATATATTCGTAACCC AATGGATTTCTATGGAAGTAACTTTGAATTGATACCATTTGGGACAGGTAGAAGAGG ATGTCCTGGTATTTTATATGGAGTGACAAATGCTGAGTTTATGTTGGCTGCTATGTTTT ATCATTTTGATTGGGAAATAGCAGATGGTAAAAAACCAGAAGAAATTGATTTGACTG AGGACTTTGGTGCTGGTTGCATTATGAAGTATCCTTTGAAATTGGTTCCTCATCTTGTT AACGATTAA
CrRedOx1	ATGGCTGATCGCGTGAAAACCGTAGGATGGGCAGCTCACGACAGCTCCGGCTTCCTC TCTCCCTTCCAATTCACTCGAAGGGCAACAGGTGAAGAAGATGTGAGGTTGAAGGTG TTGTACTGTGGTGTCTGTCACTCAGACCTTCATAACATCAAGAACGAAATGGGATTCA CCTCCTACCCTTGTGTCCCCGGGCATGAGGTTGTGGGGGAAGTGACGGAAGTGGGGA ACAAAGTAAAGAAATTCATAATTGGTGATAAAGTTGGGGTTGGATTATTCGTTGACT CATGTGGCGAATGCGAACAATGTGTGAATGATGTAGAAACCTATTGTCCCAAATTGA AAATGGCTTATTTATCCATTGATGATGATGGAACTGTGATTCAAGGAGGGTACTCAA AGGAAATGGTCATCAAAGAACGCTACGTTTTCCGGTGGCCGGAAAATCTTCCTCTAC CCGCCGGTACACCGCTTCTGGGTGCCGGTAGTACAGTTTATAGTCCAATGAAATACTA TGGACTTGATAAGTCAGGACAACATCTAGGAGTTGTTGGCCTTGGTGGACTTGGTCAT TTAGCTGTTAAATTTGCAAAGGCTTTTGGACTTAAAGTTACTGTCATTAGTACCTCTC CTAGCAAGAAGGATGAAGCCATCAATCACCTTGGTGCTGATGCATTTTTAGTTAGCA CTGATCAAGAGCAAACCCAGAAAGCAATGAGCACAATGGATGGCATAATTGACACA GTATCAGCTCCTCATGCATTGATGCCATTGTTTTCCCTATTGAAACCAAATGGGAAGC TAATTGTTGTTGGTGCACCAAATAAACCAGTTGAATTAGATATTCTATTTCTTGTCAT GGGAAGGAAAATGCTCGGAACATCTGCTGTTGGGGGAGTGAAGGAGACACAAGAGA TGATCGATTTTGCAGCAAAACATGGTATAGTTGCAGATGTAGAAGTTGTGGAAATGG AGAATGTGAACAATGCAATGGAGCGGCTTGCGAAGGGGGATGTTAGATATAGATTTG TTCTTGATATTGGGAATGCAACAGTAGCTGTCGGTTAA

CrRedOx2	ATGGAAAAGCAAGTTGAGATCCCTGAAGTAGAATTGAATTCAGGACATAAAATGCCA<br>ATTGTGGGATACGGAACATGCGTGCCAGAACCCATGCCACCGTTGGAAGAACTAACC<br>GCAATCTTCTTAGATGCAATAAAGGTTGGTTACCGGCACTTCGACACGGCTTCGAGTT<br>ACGGCACAGAGGAGGCACTTGGTAAAGCCATAGCTGAGGCTATAAACAGCGGTTTG<br>GTTAAAAGTAGGGAGGAATTCTTCATCAGTTGTAAGCTGTGGATTGAAGATGCAGAT<br>CATGACCTTATCTTGCCTGCCCTCAACCAGTCACTTCAGATTCTTGGGGTTGATTATTT<br>GGATCTATATATGATACACATGCCGGTGAGAGTGAGGAAAGGTGCTCCCATGTTCAA<br>TTATTCAAAAGAGGATTTCCTTCCATTTGACATACAAGGTACATGGAAGGCCATGGA<br>AGAGTGCAGCAAACAAGGATTGGCCAAGTCTATTGGTGTCAGCAACTACTCTGTTGA<br>AAAACTCACTAAACTCCTAGAAACCTCCACTATTCCCCCCGCCGTTAATCAGGTTGAG<br>ATGAATGTAGCTTGGCAACAGAGAAAATTGCTGCCATTTTGCAAAGAGAAAAACATT<br>CATATAACATCATGGTCCCCACTCCTATCTTATGGTGTCGCTTGGGGAAGTAATGCTG<br>TCATGGAAAATCCTGTTCTCCAACAAATTGCGGCTTCCAAAGGCAAAACTGTGGCAC<br>AGGTGGCACTAAGATGGATATACGAGCAAGGAGCAAGTCTCATTACAAGGACTTCCA<br>ACAAGGACAGAATGTTTGAAAATGTTCAAATTTTTGATTGGGAACTCAGTAAAGAAG<br>AATTGGATCAAATTCATGAAATCCCACAACGTAGGGGTACTTTAGGTGAAGAATTTA<br>TGCATCCAGAAGGACCGATCAAATCCCCAGAGGAGCTTTGGGATGGAGACTTGTAA
CrSAT	ATGCACCCCAGATGCAGATATTGTCAGAGGAACTGATTCAACCATCATCTCCGACAC<br>CCCAAACCTTGAAAACCCATAAACTTTCCCATCTTGATCAAGTTTTATTAACATGTCA<br>TATCCCTATTATTCTCTTTTATCCAAATCAATTGGACTCAAATCTCGATCGAGCCCAA<br>AGATCTGAGAATCTAAAACGATCTTTATCAACAGTGTTAACTCAATTTTACCCTTTAG<br>CCGGAAGAATCAATATAAATTCTTCCGTAGATTGTAATGATTCCGGAGTCCCTTTTCT<br>TGAAGCTCGAGTTCATTCCCAACTCTCAGAAGCAATTAAAAATGTCGCCATAGATGA<br>ACTCAATCAATACCTGCCATTCCAACCTTATCCCGGTGGGGAGGAAAGTGGGTTGAA<br>AAAAGATATCCCCTTAGCTGTAAAAATCAGTTGTTTCGAATGTGGCGGAACAGCAAT<br>TGGGGTCTGTATTTCCCACAAGATTGCCGATGCGTTGTCCTTGGCTACCTTCCTCAATT<br>CATGGACCGCAACATGCCAGGAAGAGACTGATATTGTTCAACCTAATTTCGATCTGG<br>GATCCCATCATTTTCCGCCTATGGAAAGCATCCCAGCACCTGAATTTCTACCGGATGA<br>AAATATTGTGATGAAACGGTTCGTGTTCGATAAAGAAAAATTAGAAGCTCTAAAAGC<br>CCAATTAGCTTCCTCTGCAACAGAAGTGAAGAATTCAAGTCGGGTACAAATTGTTATT<br>GCTGTTATATGGAAGCAATTCATTGACGTGACCCGGGCAAAATTCGATACCAAGAAC<br>AAATTAGTAGCAGCTCAAGCAGTGAATTTGAGATCAAGAATGAATCCACCATTTCCT<br>CAATCTGCTATGGGGAATATAGCCACAATGGCATATGCCGTTGCAGAGGAGGATAAG<br>GATTTTTCAGATCTTGTCGGTCCATTGAAAACCAGTCTTGCAAAAATTGATGATGAAC<br>ATGTTAAAGAATTACAAAAAGGAGTAACATATTTGGATTATGAAGCTGAACCACAAG<br>AATTGTTCTCTTTTAGTAGTTGGTGCAGGCTTGGGTTTTATGATTTGGATTTTGGATGG<br>GGAAAGCCTGTTTCTGTTTGTACAACAACTGTGCCTATGAAGAATTTGGTATATTTGA<br>TGGATACAAGAAATGAAGATGGAATGGAAGCATGGATCAGCATGGCTGAAGATGAG<br>ATGTCTATGCTTTCCTCTGATTTTCTTTCACTTCTGGACACTGATTTTAGCAATTGA

CrTHAS	ATGGCAATGGCTTCAAAGTCACCTTCTGAAGAAGTATATCCAGTGAAGGCATTTGGT<br>TTGGCTGCTAAGGATTCTTCTGGGCTTTTCTCTCCATTCAACTTCTCAAGAAGGGCCA<br>CAGGGGAACACGATGTGCAGCTCAAAGTATTATACTGTGGGACTTGCCAATATGACA<br>GGGAAATGAGCAAAAACAAATTTGGATTTACAAGCTATCCTTATGTTTTAGGGCATG<br>AAATTGTGGGTGAGGTAACTGAAGTTGGCAGCAAGGTGCAGAAATTCAAAGTCGGG<br>GACAAAGTGGGCGTAGCAAGCATAATTGAAACTTGTGGCAAATGTGAAATGTGTACA<br>AATGAAGTTGAAAATTACTGTCCAGAAGCAGGATCAATAGACAGCAATTACGGGGC<br>ATGTTCAAATATAGCAGTGATAAACGAGAATTTTGTCATCCGTTGGCCTGAAAATCTT<br>CCTTTGGATTCTGGTGTTCCTCTTCTATGTGCAGGAATCACGGCTTATAGTCCCATGA<br>AACGTTATGGACTTGATAAACCTGGAAAACGTATCGGCATAGCCGGTCTAGGAGGAC<br>TTGGACATGTAGCTCTTAGATTTGCCAAAGCTTTTGGGGCTAAGGTGACAGTGATTAG<br>TTCTTCACTTAAGAAAAAACGTGAAGCCTTTGAGAAATTCGGAGCAGATTCTTTCTTG<br>GTCAGCAGTAATCCAGAAGAAATGCAGGGTGCAGCAGGAACATTGGATGGGATCAT<br>AGACACTATACCAGGGAATCACTCTCTTGAGCCACTCCTTGCTTTATTGAAGCCTCTT<br>GGGAAGCTTATCATTTTAGGTGCACCAGAAATGCCCTTTGAGGTTCCCGCTCCTTCCC<br>TGCTTATGGGTGGAAAAGTAATGGCTGCCAGTACTGCTGGGAGTATGAAGGAAATAC<br>AAGAGATGATTGAATTTGCAGCAGAACACAACATAGTAGCAGATGTGGAGGTTATCT<br>CTATTGACTATGTGAACACTGCAATGGAGCGCCTTGATAACTCTGATGTGAGATATCG<br>TTTCGTGATTGATATAGGGAACACTCTGAAATCAAATTAA
CrAS	ATGGATCAGCTGATGAACTTCTCTCTCACCTCTCCCATTTTCCTTCTTCTCTCCTCCTT<br>ATTTCTCATCATCTTACTTAACAAACTGATGAGAGGTAACAAAATCCAAAAGGGCAA<br>AAAACTACCACCAGGTCCAAAAAAAATTGCCATAATAGGGAACCTCCATCAAATGGT<br>AGGTTCACTTCCTCATCGTGTTCTAAACAATTTAGCAGAAAAATATGGACCGATCATG<br>CACTTGCGAATCGGCCAACTTTCAGCCGTTATTATATCATCAGCAGAAAAAGCGAAA<br>GAAATATTAAACACTCATGGTGTTCGTGTCGCGGACAGGCCACAAACTACTGTGGCT<br>AAAATCATGTTGTATAATAGTTTAGGTGTCACTTTTGCTCCCTATGGTGATTACCTAA<br>AACAATTGCGTCAAATTTATGCGATGGAACTTCTTAGCCCTAAAACAGTGAAGTCCTT<br>TTGGACAATTATGGACGATGAGCTTTCCACAATGATAACCTCAATTAAATCTGAAGTT<br>GGTCAACCAATGATTTTACATGATAAAATGATGACTTATTTATATGCTATGCTTTGTA<br>GAGCTACAGTTGGTAGCGTATGTAATGGACGAGAGACTCTAATTATGGCAGCTAAAG<br>AAACGTCAGCTCTTTCTGCTTCTATTAGGATTGAAGATTTATTTCCTTCGGTGAAAAT<br>ACTTCCTGTAATTAGTGGATTGAAGTCAAAATTAACTAACTTGTTGAAAGAACTTGAT<br>ATTGTTCTTGAGGATATTATTAGTGCTCGTGAGAAGAAATTATTAAGCCAGCCCCAGC<br>AGCCATTAATGTTGGATGAAGAAGATATGTTGGGAGTTCTCCTCAAGTACAAAAATG<br>GAAAGGGAAATGATACTAAATTTAGAGTCACCAACAATGACATCAAGGCCATCGTTT<br>TTGAACTGATTTTGGCTGGAACTCTAAGTTCAGCAGCTATAGTAGAATGGTGTATGTC<br>AGAATTGATGAAAAACCCTGAATTATTGAAGAAGGCACAAGATGAAGTTAGGCAAG<br>TTCTAAAGGGTAAGAAAACAATAAGTGGAAGTGATGTTGGTAAATTGGAATATGTTA<br>AAATGGTGGTTAAGGAATCAGTGAGATTACATCCTCCAGCTCCCCTTTTATTTCCAAG<br>AGAATGTAGGGAAGAATTTGAGATAGATGGAATGACCATACCTAAAAAATCTTGGGT<br>GATTATAAACTACTGGGCAATTGGAAGAGATCCCAAAATTTGGCCCAATGCTGATAA<br>GTTTGAGCCTGAGAGATTCAGTAATAACAACATTGATTTCTATGGAAGCAATTTTGAG<br>TTGATACCATTTGGTGCTGGAAGAAGGGTTTGTCCTGGAATATTATTTGGAACAACTA<br>ATGTTGAGCTTTTGCTTGCTGCATTTCTCTTTCATTTTGATTGGGAACTTCCTGGAGGC<br>ATGAAACCAGAAGAATTAGATATGAATGAGTTATTTGGTGCTGGTTGCATTAGAGAA<br>AATCCTTTGTGTCTCATTCCTAGCATCTCAACTGTAGTTGAAGGAAACTAA

SlUbi10_Pr	GGAGGTCAACTACCCCAATTTAAATTTTATTTGATTAAGATATTTTTATGGACCTACT<br>TTATAATTAAAAATATTTTCTATTTGAAAAGGAAGGACAAAAATCATACAATTTTGGT<br>CCAACTACTCCTCTCTTTTTTTTTTTGGCTTTATAAAAAAGGAAAGTGATTAGTAATA<br>AATAATTAAATAATGAAAAAAGGAGGAAATAAAATTTTCGAATTAAAATGTAAAAG<br>AGAAAAAGGAGAGGGAGTAATCATTGTTTAACTTTATCTAAAGTACCCCAATTCGAT<br>TTTACATGTATATCAAATTATACAAATATTTTATTAAAATATAGATATTGAATAATTT<br>TATTATTCTTGAACATGTAAATAAAAATTATCTATTATTTCAATTTTTATATAAACTAT<br>TATTTGAAATCTCAATTATGATTTTTTAATATCACTTTCTATCCATGATAATTTCAGCT<br>TAAAAAGTTTTGTCAATAATTACATTAATTTTGTTGATGAGGATGACAAGATTTCGGT<br>CATCAATTACATATACACAAATTGAAATAGTAAGCAACTTGATTTTTTTTCTCATAAT<br>GATAATGACAAAGACACGAAAAGACAATTCAATATTCACATTGATTTATTTTTATATG<br>ATAATAATTACAATAATAATATTCTTATAAAGAAAGAGATCAATTTTGACTGATCCA<br>AAAATTTATTTATTTTTACTATACCAACGTCACTAATTATATCTAATAATGTAAAACA<br>ATTCAATCTTACTTAAATATTAATTTGAAATAAACTATTTTTATAACGAAATTACTAA<br>ATTTATCCAATAACAAAAAGGTCTTAAGAAGACATAAATTCTTTTTTTGTAATGCTCA<br>AATAAATTTGAGTAAAAAAGAATGAAATTGAGTGATTTTTTTTTAATCATAAGAAAA<br>TAAATAATTAATTTCAATATAATAAAACAGTAATATAATTTCATAAATGGAATTCAAT<br>ACTTACCTCTTAGATATAAAAAATAAATATAAAAATAAAGTGTTTCTAATAAACCCG<br>CAATTTAAATAAAATATTTAATATTTTCAATCAAATTTAAATAATTATATTAAAATAT<br>CGTAGAAAAAGAGCAATATATAATACAAGAAAGAAGATTTAAGTACAATTATCAACT<br>ATTATTATACTCTAATTTTGTTATATTTAATTTCTTACGGTTAAGGTCATGTTCACGAT<br>AAACTCAAAATACGCTGTATGAGGACATATTTTAAATTTTAACCAATAATAAAACTA<br>AGTTATTTTTAGTATATTTTTTTGTTTAACGTGACTTAATTTTTCTTTTCTAGAGGAGC<br>GTGTAAGTGTCAACCTCATTCTCCTAATTTTCCCAACCACATAAAAAAAAAAATAAAG<br>GTAGCTTTTGCGTGTTGATTTGGTACACTACACGTCATTATTACACGTGTTTTCGTATG<br>ATTGGTTAATCCATGAGGCGGTTTCCTCTAGAGTCGGCCATACCATCTATAAAATAAA<br>GCTTTCTGCAGCTCATTTTTTCATCTTCTATCTGATTTCTATTATAATTTCTCTGAATTG<br>CCTTCAAATTTCTCTTTCAAGGTTAGAATTTTTCTCTATTTTTTGGTTTTTGTTTGTTTA<br>GATTCTGAGTTTAGTTAATCAGGTGCTGTTAAAGCCCTAAATTTTGAGTTTTTTTCGGT<br>TGTTTTGATGGAAAATACCTAACAATTGAGTTTTTTCATGTTGTTTTGTCGGAGAATG<br>CCTACAATTGGAGTTCCTTTCGTTGTTTTGATGAGAAAGCCCCTAATTTGAGTGTTTTT<br>CCGTCGATTTGATTTTAAAGGTTTATATTCGAGTTTTTTTCGTCGGTTTAATGAGAAGG<br>CCTAAAATAGGAGTTTTTCTGGTTGATTTGACTAAAAAAGCCATGGAATTTTGTGTTT<br>TTGATGTCGCTTTGGTTCTCAAGGCCTAAGATCTGAGTTTCTCCGGTTGTTTTGATGAA<br>AAAGCCCTAAAATTGGAGTTTTTATCTTGTGTTTTAGGTTGTTTTAATCCTTATAATTT<br>GAGTTTTTTCGTTGTTCTGATTGTTGTTTTTATGAATTTTGCAG

SlUbi10_Te	GCTTGTTGTGGTTGTCTGGTTGCGTCTGTTGCCCGTTGTCTGTTGCCCATTGTGGTGGT<br>TGTGTTTGTATGATGGTCGTTAAGGATCATCAATGTGTTTTCGCTTTTTGTTCCATTCT<br>GTTTCTCATTTGTGAATAATAATGGTATCTTTATGAATATGCAGTTTGTGGTTTCTTTT<br>CTGATTGCAGTTCTGAGCATTTTGTTTTTGCTTCCGTTTACTATACCACTTACAGTTTG<br>CACTAATTTAGTTGATATGCGAGCCATCTGATGTTTGATGATTCAAATGGCGTTTATG<br>TAACTCGTACCCGAGTGGATGGAGAAGAGCTCCATTGCCGGTTTGTTTCATGGGTGG<br>CGGAGGGCAACTCCTGGGAAGGAACAAAAGAAAAACCGTGATACGAGTTCATGGGT<br>GAGAGCTCCAGCTTGATCCCTTCTCTGTCGATCAAATTTGAATTTTTGGATCACGGCA<br>GGCTCACAAGATAATCCAAAGTAAAACATAATGAATAGTACTTCTCAATGATCACTT<br>ATTTTTAGCAAATCAGCAATTGTGCATGTCAAATGATTTCGGTGTAAGAGAAAGAGT<br>TGATGAATCAAAATATCTGTAGCTGGATCAAGAATCTGAGGCAGTTGTATGTATCAA<br>TGATCTTTCCGCTACAATGATGTTAGCTATCCGAGTCAAATTGTTGTAGAATTGCATA<br>CTTCGGCATCACATTCTGGATGACATAATAAATAGGAAGTCTTCAGATCCCTAAAAA<br>ATTGAGAGCTAATAACATTAGTCCTAGATGTAACTGGGTGACAACCAAGAAAGAGAC<br>ATGCAAATACTACTTTTGTTTGAAGGAGCATCCCTGGTTTGACATATTTTTTCTGAAT<br>ATCAAACTTTGAAACTCTACCTAGTCTAATGTCTAACGACAGATCTTACTGGTTTAAC<br>TGCAGTGATATCTACTATCTTTTGGAATGTTTTCTCCTTCAGTTATACATCAAGTTCCA<br>AGATGCAGGTGTGCTTGATTGATGTACATGGCTGTGAGAAGTGCATCCTGATGTTCA<br>GATGATGGTTCATTCTAATGTCTTTTCCTTCAATCAGTTTTCTCAGTCTGACTTAGCTT<br>GTTTCATCTGCATGTTTGAATGTTCGTTTACTCATAGTAATTGCATTTTTGTAGCAGAA<br>CATATCATTGGTCATGGTTTCAACTGTGCGCGAGTCTTATGCTTATTCAAACTAGGAA<br>AGCCTCCGTCTAGAGGGTACACGAGTTGTTGCTCTGTGTGCGTCAGTCCATAGTATTA<br>ATCTTGCTAGTTGTAGTATATTGTTTATGTGGACTCGGAATTCATCATATGCTCCTTCT<br>TTGCATCAAGTAAGGCAAGGTAATGTATAGAAGCTTTTTAACTCTTTCATGGAAGCTG<br>GCCTTTGCCAGCATACCATCCAGAAGATATCAACCCTGCATCTTGGCTGCCG

Table S3. Sequences of forward and reverse primers used in this investigation. Underlined sequences are the cloning overhangs.

Gene	Forward primer	Reverse primer
CrSGD	TTGCGGTCTCTAATGGGTTCTAAGGATGATCAA	GCAAGGTCTCTAAGCTTAATACTTCTGTTTCTTAA
CrGS	TTGCGGTCTCTAATGGCCGGAGAAACAACCAAA	GCAAGGTCTCTAAGCTTATTCCTCAAATTTCAATG
CrGO	TTGCGGTCTCTAATGGAGTTTTCTTTCTCCTCA	GCAAGGTCTCTAAGCTTAATCGTTAACAAGATGAGGAA
CrRedOx1	TTGCGGTCTCTAATGCTGATCGCGTGAAAACCGT	GCAAGGTCTCTAAGCTTAACCGACAGCTACTGTTG
CrRedOx2	TTGCGGTCTCTAATGGAAAAGCAAGTTGAGATC	GCAAGGTCTCTAAGCTTACAAGTCTCCATCCCAAA
CrSAT	TTGCGGTCTCTAATGCGGGTCTCTAATGCACCCCAGATGCAGATAT	GCAAGGTCTCTAAGCTCAATTGCTAAAATCAGT
CrTHAS	TTGCGGTCTCTAATGGCAATGGCTTCAAAGTCAC	GCAAGGTCTCTAAGCTTAATTTGATTTCAGAGTGTTC
CrAS	TTGCGGTCTCTAATGGATCAGCTGATGAACTTCTCTC	GCAAGGTCTCTAAGCTTAGTTTCCTTCAACTACAGTT
SlUbi10_Pr	TGGTCTCGGGAGGTCAACTACCCCAATTTAAAT	TGGTCTCGCATTCTGCAAAATTCATAAAAACAACAA
SlUbi10_Te	GTGGTCTCGGCTTGTTGTGGTTGTCTGGTTGCGTC	GTGGTCTCGAGCGCGGCAGCCAAGATGCAGGGTTGATAT

Table S4. MRM transitions, collision energy and retention times of the compounds analyzed in this study.

	Quantifier ion		*Qualifier ion 1*	*Qualifier ion 2*	
Compound	*Q1 m/z*	*CE (eV)*	*Q2 m/z*	*Q2 m/z*	*Retention time*
Serpentine	349.18	55	205.9	262.9	4.95
4-Fluoro alstonine	367.18	55	223.9	280.9	5.20
5-Fluoro alstonine	367.18	55	223.9	280.9	5.18
6-Fluoro alstonine	367.18	55	223.9	280.9	5.23
7-Fluoro alstonine	367.18	55	223.9	280.9	5.05
7-Chloro alstonine	383.18	55	239.9	296.9	5.68
Stemmadenine acetate	397.2	41	168.2	227.9	4.16
4-Fluoro stemmadenine acetate	415.20	41	186.2	245.2	4.71
5-Fluoro stemmadenine acetate	415.20	41	186.2	245.2	4.64
6-Fluoro stemmadenine acetate	415.20	41	186.2	245.2	4.47
7-Fluoro stemmadenine acetate	415.20	41	186.2	245.2	4.39
Tetrahydroalstonine	353.20	25	144.2	122.2	4.77
6-methyl tetrahydroalstonine	367.2	25	158.0	235.0	5.39
7-methoxy tetrahydroalstonine	383.2	25	174.0	251.0	4.84
Precondylocarpine acetate	395.2	22	168.9	227.0	3.85
4-fluoro precondylocarpine acetate	413.2	22	186.9	245.0	3.95
5-fluoro precondylocarpine acetate	413.2	22	186.9	245.0	4.21
6-fluoro precondylocarpine acetate	413.2	22	186.9	245.0	4.46
7-fluoro precondylocarpine acetate	413.2	22	186.9	245.0	4.38
Akuammicine	323.2	20	291.1	182.1	4.22
6-Methyl akuammicine	337.2	20	305.1	196.1	5.20
7-Methyl akuammicine	337.2	20	305.1	196.1	5.60
4-fluoro akuammicine	341.2	20	309.1	200.1	4.21
5-fluoro akuammicine	341.2	20	309.1	200.1	4.49
6-fluoro akuammicine	341.2	20	309.1	200.1	4.70
7-fluoro akuammicine	341.2	20	309.1	200.1	5.00
